# Category-Selectivity in Human Visual Cortex Follows Cortical Topology: A Grouped icEEG Study

**DOI:** 10.1371/journal.pone.0157109

**Published:** 2016-06-07

**Authors:** Cihan Mehmet Kadipasaoglu, Christopher Richard Conner, Meagan Lee Whaley, Vatche George Baboyan, Nitin Tandon

**Affiliations:** 1 Vivian Smith Department of Neurosurgery, University of Texas Medical School at Houston, Houston, TX, United States of America; 2 Memorial Hermann Hospital, Texas Medical Center, Houston, TX, United States of America; Centre de Neuroscience Cognitive, FRANCE

## Abstract

Neuroimaging studies suggest that category-selective regions in higher-order visual cortex are topologically organized around specific anatomical landmarks: the mid-fusiform sulcus (MFS) in the ventral temporal cortex (VTC) and lateral occipital sulcus (LOS) in the lateral occipital cortex (LOC). To derive precise structure-function maps from direct neural signals, we collected intracranial EEG (icEEG) recordings in a large human cohort (*n* = 26) undergoing implantation of subdural electrodes. A surface-based approach to grouped icEEG analysis was used to overcome challenges from sparse electrode coverage within subjects and variable cortical anatomy across subjects. The topology of category-selectivity in bilateral VTC and LOC was assessed for five classes of visual stimuli—faces, animate non-face (animals/body-parts), places, tools, and words—using correlational and linear mixed effects analyses. In the LOC, selectivity for living (faces and animate non-face) and non-living (places and tools) classes was arranged in a ventral-to-dorsal axis along the LOS. In the VTC, selectivity for living and non-living stimuli was arranged in a latero-medial axis along the MFS. Written word-selectivity was reliably localized to the intersection of the left MFS and the occipito-temporal sulcus. These findings provide direct electrophysiological evidence for topological information structuring of functional representations within higher-order visual cortex.

## Introduction

Visual object recognition is a ubiquitous feature in our day-to-day lives, enabling us to recognize the faces of our loved ones, find a favorite snack in the grocery aisle, and even read the words on this page. Achieved with rapidity and accuracy, object recognition appears nearly effortless. Yet the apparent automaticity with which we perform this feat belies its underlying neural complexity, and damage to any part of the network of cortical regions involved may produce debilitating deficits—such as visual agnosias (e.g. face-blindness)—that can seriously affect social or vocational life [1, 2].

Extensive human and non-human primate research has identified putative higher-order visual areas in the ventral temporal and lateral occipital cortical complexes (VTC and LOC, respectively), which are believed to mediate object recognition via the activity of distinct neuronal clusters that differentially and selectively activate to specific categories of visual stimuli (e.g. faces/places/animals/tools/words) [3–18]. However, the functional and organizational principles of the VTC and LOC continue to remain a topic of debate. This is largely due to the considerable variability in anatomical location and spatial relation of different category specific regions reported in subjects, both within and across studies [19–22].

Recently, advances in functional, structural, and anatomical neuroimaging have begun to yield new insights into structure-function relationships of the VTC and LOC [23]. Specifically, in the VTC, the mid-fusiform sulcus (MFS) has been revealed to predict lateral-to-medial transitions in receptor and cyto-architectonics, white-matter connectivity, and large-scale functional maps (e.g. animacy maps, eccentricity bias); while in the LOC, dorso-ventral transitions in large-scale functional maps appear to be arranged around the lateral occipital sulcus (LOS). Further comparisons between the MFS/LOS and the relative organization of category-selective regions have revealed that these smaller-scale functional representations also align with the same sulcal landmarks [21, 22, 24–41].

Taken together, these findings suggest that these anatomical landmarks—the MFS and LOS—may provide a structural framework for the organization of higher-order visual representations, in which opposing sides of these sulci contain neural hardware for processing distinct classes of visual information (foveal vs. peripheral, animate vs. inanimate, face vs. place) [23]. Importantly, smaller-scale functional representations appear to be nested within larger-scale representations, such that visual information processing is organized in a way that mirrors the hierarchical organization of human conceptual knowledge [23]. Concrete (i.e. basic-level) categorical information is embodied at smaller spatial scales, via category-selective regions, while abstract (i.e. superordinate-level) categorical information is reflected at larger spatial scales [22, 23, 42]. For example, lateral to the MFS, face and body-part selective regions (basic-level information) are localized adjacent to each other, and converge within animate representations (superordinate-level) of large-scale animacy maps [21, 27, 31, 33, 37]. Similarly, medial to the MFS, tool and place-selective regions converge within large-scale inanimate representation [14, 31, 39]. This hierarchical structuring of visual information might explain how the VTC and LOC may be biologically optimized to achieve rapid object recognition and categorization [23].

Notably, given the spatial constraints of the VTC and LOC (i.e. a 2D cortical sheet), different functional maps (e.g. animacy and eccentricity bias) appear to be organized on the same spatial gradients around the MFS and LOS, respectively [23]. However, the correspondence between different functional maps is not necessarily one-to-one. For instance, in addition to animacy distinctions, the MFS also predicts medio-lateral transitions in eccentricity bias maps (i.e. peripheral vs. foveal representations, respectively) [33, 43]. And while place (inanimate and peripheral) and face (animate and foveal) stimuli engage medial and lateral regions of the MFS, respectively, word stimuli (inanimate and foveal) selectively engage regions lateral to face-selective regions, in the vicinity of the occipitotemporal sulcus (OTS) [44–46].

While fMRI studies have made great strides towards understanding the organization of these visual areas, the spatio-temporal resolution and indirect nature of hemodynamic measures prevents a definitive assessment of their functional topography [47, 48]. Although newer analytic approaches have been developed to address the limitations of traditional localization-based techniques (e.g. multivariate pattern analysis) [49–53], their relationship to the underlying neural population activity has not been validated in humans [54, 55]. Human intracranial EEG (icEEG) recordings provide high spatiotemporal resolution neural recordings and offer a unique opportunity to validate hypotheses of VTC and LOC organization [56–58].

Despite recent work, a comprehensive icEEG investigation into the topology of VTC and LOC category-selectivity remains lacking (for review see [59]). This is due largely to challenges arising from spatially variable and sparse electrode coverage within subjects. The discrete and clinically directed implantation of electrodes precludes evaluation of both small and large-scale functional organization in any single individual, requiring the combination of data across a large number of subjects to achieve adequate cortical coverage. However, current approaches for the spatial co-registration of datasets across individuals (e.g. affine/volumetric normalizations) are unable to preserve the topological alignment of homologous functional regions, due to the highly folded (nonlinear) cortical geometry [60]. As a result, prior icEEG studies have focused more on evaluating the functional properties of category-selective regions, but not their topological organization within the VTC and LOC (but see [59]) [3, 17, 61–70].

Recently, new methodological advances have introduced surface-based normalization strategies for grouping icEEG data [60, 71, 72], which provide computationally efficient methods to correct for inter-subject anatomical variability and sparse-sampling [73]. In the current study, we utilized one such surface-based grouped icEEG approach [60] to investigate VTC and LOC category tuning across a large patient cohort (*n* = 26); using data collected during the visual naming of living (faces, animals, and body-parts) and non-living (tools and places) stimuli, as well as during a wordstem completion task.

If models of visual information structuring are accurate [23], we expect electrodes with selectivity for living (face and other animate) stimuli to be localized lateral to the MFS in the VTC, and ventral to the LOS in the LOC. Also, lateral to the MFS (in the left VTC), we expect word-selective electrodes to co-localize with face-selective electrodes in regions biased toward foveal representation. In contrast, electrodes with selectivity for non-living stimuli (tools and places) should localize medial to the MFS in the VTC and superior to the LOS in the LOC. Importantly, we expect the relative spatial arrangements of category-selective electrodes (within individuals) to be preserved within larger-scale functional representations *at the group level* (across individuals).

To test this hypothesis, we generated topologically precise population-level maps of icEEG data [60], and directly evaluated whether: 1) large-scale functional maps (e.g. animacy: living vs. non-living) emerge from the relative arrangements of distinct category-selective regions in the VTC and LOC; and 2) that transitions in multi-scale functional maps are preserved around specific sulcal landmarks (e.g. MFS and LOS, respectively). We found that, in the LOC, selectivity for living and non-living stimuli is arranged along the LOS about a ventral-to-dorsal axis. In the VTC, living and non-living stimuli are arranged along the MFS about a lateral-to-medial axis. Furthermore, in the left VTC, word-selectivity is reliably predicted by the intersection of the anterior MFS and the occipitotemporal sulcus, and is interspersed with other foveally represented categories. These results were consistent at both the individual and population-level, and provide direct evidence for structure-function coupling in the VTC and LOC from electrophysiological data in humans.

## Materials and Methods

Data were collected from 26 subjects (16 females, mean age 33 ± 11 years, mean IQ 100 ± 11) undergoing left (LH, *n* = 16) or right hemispheric (RH, *n =* 10) subdural electrode (SDE) implantation (Table 1). All experimental procedures were reviewed and approved by the Committee for the Protection of Human Subjects (CPHS) of the University of Texas Health Science Center at Houston as Protocol Number: (HSC-MS-06-0385), and written informed consent was obtained from all subjects.

**Table 1:** Patient demographics.

ID	Age	Sex	IQ	Hand	# SDEs: VTC	# SDEs: LOC	Wordstem Task
L1	21	M	97	R	10	1	Y
L2	39	M	100	R	3	1	Y
L3	30	F	100	R	1	4	Y
L4	20	F	97	R	1	1	Y
L5	42	F	107	R	9	3	Y
L6	51	F	92	R	4	0	Y
L7	42	M	104	R	4	0	Y
L8	23	M	91	R	9	7	Y
L9	29	F	84	R	8	3	N
L10	23	M	101	L	3	0	Y
L11	34	F	97	R	3	0	Y
L12	21	F	78	R	5	0	N
L13	31	F	124	R	5	0	Y
L14	28	F	95	R	8	12	Y
L15	25	F	101	R	13	9	Y
L16	18	F	75	R	8	7	N
R1	62	F	93	R	3	0	N
R2	27	M	112	R	4	10	N
R3	40	M	94	R	5	0	N
R4	28	F	107	L	8	2	N
R5	49	M	111	R	4	0	N
R6	42	F	118	L	4	0	N
R7	29	M	102	L	4	0	N
R8	36	M	103	A	22	13	N
R9	43	F	108	R	5	7	N
R10	46	F	98	R	5	3	N

### Experimental design

**Fig 1:**
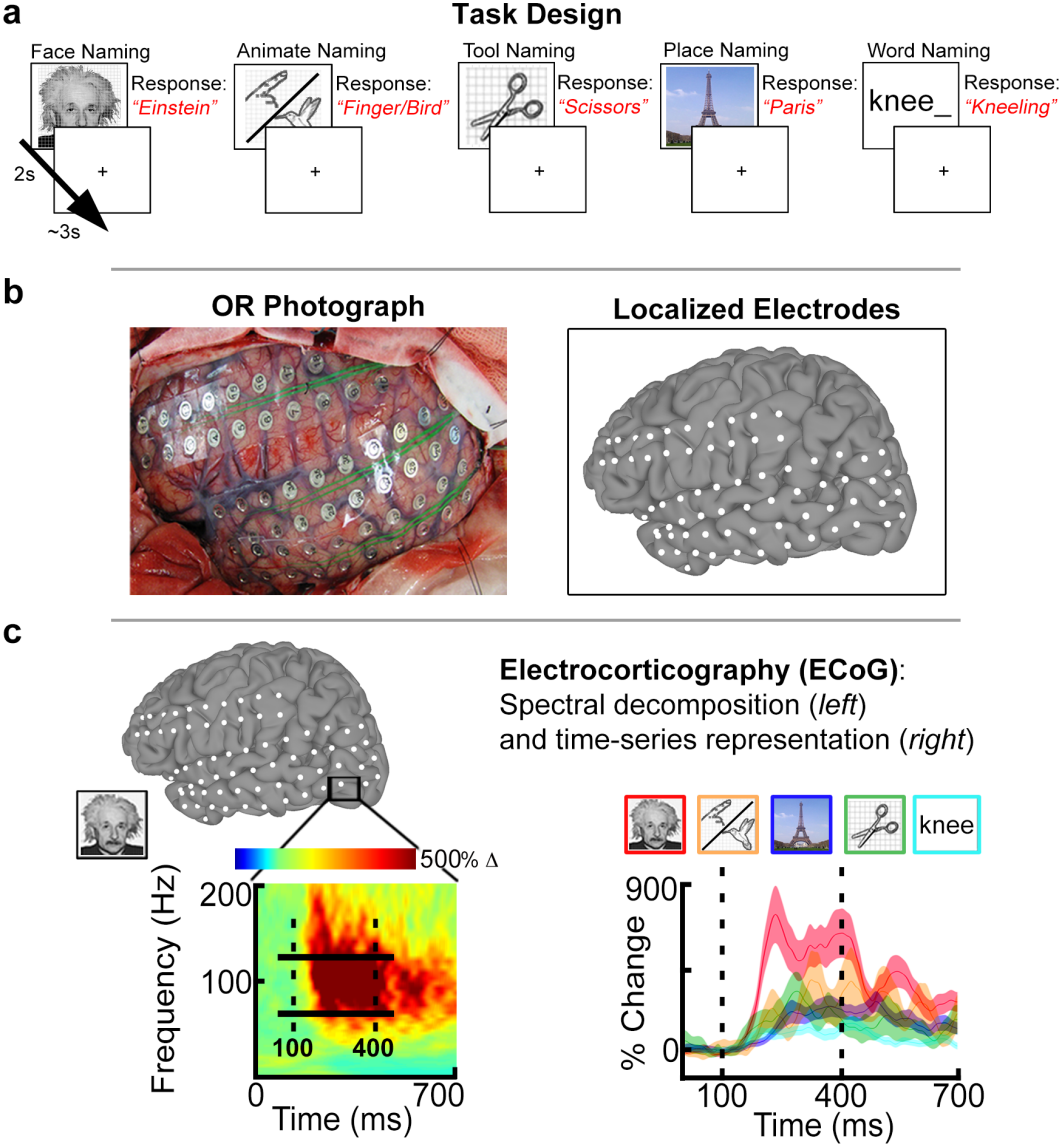
Experimental design and analysis. (A) Patients performed naming of 5 stimulus categories: faces, animate non-face (animals/body parts), places, tools, and words. Images were presented for 2 seconds followed by a jittered 3s inter-stimulus interval. Exemplar responses are indicated in red text. (B) Subjects were implanted with subdural electrodes (SDEs) in either the left (LH) or right hemisphere (RH). SDEs were localized to subject cortical surface models and represented as spheroids (white) centered on electrode coordinates. (C) Cortical activity was measured using electrocorticography (ECoG). (*Left)* ECoG data were spectrally decomposed to obtain percent-power change in the broadband gamma frequency range (BGA, 60 to 120 Hz; solid horizontal bars) relative to a pre-stimulus baseline window (-700 to -200 ms). The spectrogram depicts the response during face naming for a single SDE (black box) in the inferior occipital gyrus. (*Right)* For the same SDE, time-series representations of BGA are plotted per category. BGA for faces (red) is greatest compared to animate (orange), place (blue), tool (green), and word (cyan) stimuli. Shadings denote 1 SEM. Vertical dashed lines denote the time window (100 to 400ms; stimulus onset @ *t* = 0 ms) used to compute *d’* sensitivity indices.

Subjects participated in a visual confrontation-naming task using 5 categories [74]: famous faces, animate non-face (animals and body-parts; hereafter referred to as “animate”), famous places, tools, and word stimuli (Fig 1a; ~80 to 120 stimuli per category).

Pictorial stimuli (face, animate, place, tool) were displayed at eye-level on a 15” LCD screen placed at 2 feet from the patient (2000 ms on screen, jittered 3000 ms inter-stimulus interval; 500 x 500 pixel image size, ~10.8° x 10.8° of visual angle, with a grid overlay on 1300 x 800 pixel white background, ~28.1° x 17.3° of visual angle). Subjects were instructed to overtly name the stimuli during the experiment. Face stimuli consisted of gray-scale, real images of famous individuals shown in frontal view (celebrities, politicians, and historical figures). Place stimuli consisted of color, real images of famous landmarks (e.g. Eiffel tower, Grand Canyon). Animate and tool stimuli were from the Snodgrass and Vanderwart object pictorial set [75]. Word stimuli were presented as partial word stems (e.g. “kne_”) to which subjects were instructed to respond with the first action word that came to mind (e.g. “kneeling”). Words consisted of black, lower-case text (2000 ms on screen, jittered 3000 ms inter-stimulus interval; font height of 100 pixels, Calibri font type, ~2.1° of visual angle) centered on a 1300 x 800 pixel white background.

For each category, images were randomly selected from our database and never repeated, so each subject saw a unique sequence of images. All subjects in both right and left hemispheric cohorts participated in the visual naming tasks with pictorial stimuli. However, given the strong hemispheric bias associated with word reading [13, 76–78], the word-naming task was only performed in the left hemispheric cohort. Due to clinical time constraints, 12 of 16 subjects in the left hemisphere cohort completed the word-naming task. A transistor-transistor logic pulse triggered by the stimulus presentation software (Python v2.7) at stimulus onset was recorded as a separate input during the experiments to time lock all trials during all tasks [79].

### Cortical surface models

Pre-implantation anatomical MRI scans were collected using a 3T whole-body MR scanner (Philips Medical Systems, Bothell WA) equipped with a 16-channel SENSE head coil. Anatomical images were collected using magnetization-prepared 180-degree radio-frequency pulses and rapid gradient-echo (MP-RAGE) sequence, optimized for gray-white matter contrast, with 1 mm thick sagittal slices and an in-plane resolution of 0.938 x 0.938 mm [80]. Cortical surface models (Fig 1b) were reconstructed using FreeSurfer software (v5.1) [81], and imported to SUMA for visualization [73].

### Electrode localization and selection criteria

A total of 3506 SDEs (LH *n* = 2101; RH *n* = 1386) were implanted (PMT Corporation; top-hat design; 3 mm diameter contact with cortex) using previously published techniques [58]. 933 SDEs (LH *n* = 482; RH *n* = 451) were excluded due to proximity to seizure onset sites, inter-ictal spikes, or 60 Hz noise. The remaining 2573 SDEs (LH *n* = 1619, RH *n* = 935) were localized to cortical surface models using intra-operative photographs and an in-house recursive grid partitioning technique [82].

**Fig 2:**
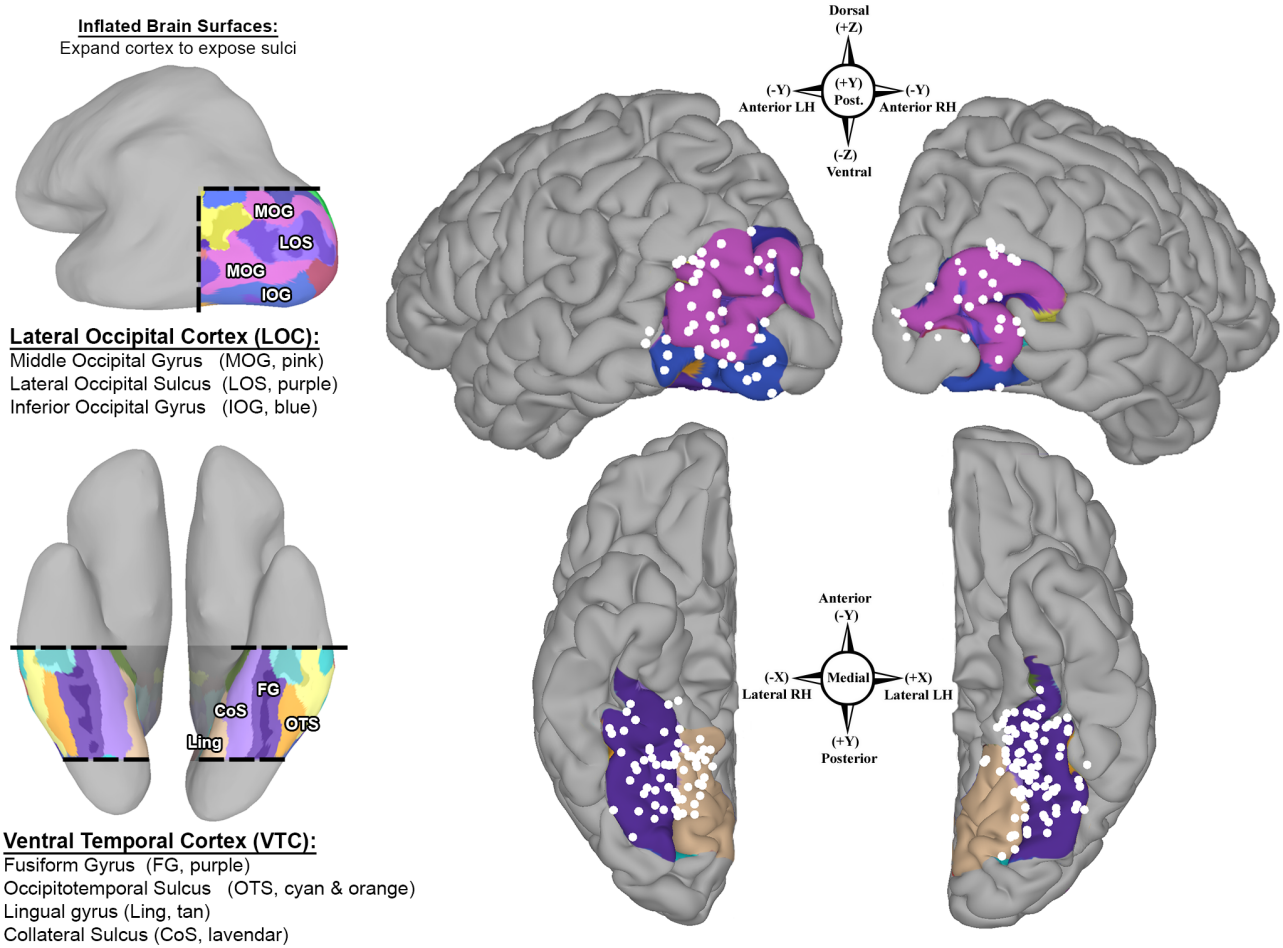
Population coverage of higher-level visual cortex. Bilateral group-electrode coverage maps depict subdural electrodes (SDEs, white spheres) from all subjects (*n* = 26 subjects; LH *n* = 16; RH *n* = 10) on a common cortical surface (MNI N27 template brain aligned to Talairach coordinate space). A total of 3506 SDEs were implanted, from which 242 SDEs were localized to the lateral occipital cortex (LOC, top; LH *n* = 48, RH *n* = 35) and the ventral temporal cortex (VTC, bottom; LH *n* = 94, RH *n* = 64). Spatial transformation of individual SDE coordinates to Talairach space was performed in a surface-based fashion. Compass points denote SDE coordinates (Talairach space) and direction in each region. The VTC and LOC, and their respective boundaries, are highlighted (*Left*) using FreeSurfer’s automated gyral and sulcal parcellations and depicted on an inflated brain surface. The LOC consists of the middle occipital (MOG, pink) and inferior occipital gyri (IOG, blue), and lateral occipital sulcus (light purple, between IOG and MOG). The VTC consists of the fusiform gyrus (purple), occipitotemporal sulcus (cyan and orange), lingual gyrus (tan) and the posterior transverse collateral sulcus (teal).

Using anatomical criteria, we identified all SDEs localized to the VTC and LOC for each individual in native anatomical space. The VTC includes the fusiform gyrus—bounded laterally by the occipitotemporal sulcus, medially by the collateral sulcus and anterior lingual gyri, posteriorly by the posterior transverse collateral sulcus, and anteriorly by the anterior tip of the mid-fusiform sulcus (MFS) [23]. The LOC includes the middle and inferior occipital gyri—bounded dorsally by the transverse occipital sulcus, ventrally by the occipitotemporal sulcus, posteriorly by the occipital pole, and anteriorly by the posterior superior temporal sulcus, as well as the posterior aspects of the inferior and middle temporal gyri (Fig 2) [21, 38, 39, 46].

To enable a population-level evaluation of category-selective topology, individual subject SDE coordinates were mapped to a standardized cortical surface (MNI N27 template brain aligned to Talairach coordinate space) using a surface-based normalization strategy (rather than affine or non-linear volumetric transformations) [60, 73, 83–85], to maximize the overlap between topologically and functionally homologous regions across subjects [86–88]. This surface-based normalization approach is detailed in an earlier publication from our group [60, 82]. Briefly, surface-based representations of electrode coverage are generated with respect to each subject’s cortical model, using geodesic metrics to correct for local gyral and sulcal folding patterns. Individual electrode datasets are subsequently normalized to a standardized cortical surface mesh, using SUMA’s surface-based normalization strategy, to enable a one-to-one correspondence between anatomical locations across subjects [60]. A total of 159 SDEs (LH *n* = 94, RH *n* = 64) were localized to the VTC and 83 SDEs (LH *n* = 48, RH *n* = 35) to the LOC (Fig 2, Table 1).

### Electrocorticographic (ECoG) processing

In 14 subjects, ECoG data were collected at 1000 Hz using NeuroFax software (Nihon Kohden, Tokyo, Japan) (bandwidth 0.15–300 Hz). The other 12 subjects underwent ECoG data collection at 2000 Hz (bandwidth 0.1–750 Hz) using the NeuroPort recording system (Blackrock Microsystems, Salt Lake City, UT). Electrodes were referenced to a common average of all electrodes in a given subject, except for those with 60 Hz noise or epileptiform activity when initially referenced to an artificial 0V [89]. All electrodes with greater than 10 dB of noise in the 60 Hz band, inter-ictal epileptiform discharges, or localized to sites of seizure onset were excluded.

To focus only on perceptual processes, analyses were restricted to a period 100–400 ms after stimulus presentation [59, 66, 90, 91]. For all ECoG data, analyses were performed by first bandpass filtering raw ECoG data into the broadband gamma frequency range (60–120 Hz, following removal of 60Hz line noise and its harmonics; IIR Elliptical Filter, 30 dB sidelobe attenuation). A Hilbert transform was applied and the analytic amplitude was smoothed (Savitzky-Golay FIR, 5^th^ order, frame length of 155 samples; Matlab 2013b, Mathworks, Natick, MA) to estimate the time course of broadband gamma activity (BGA) [79]. BGA was utilized in our analyses as it has been demonstrated to provide precise estimates of task-specific cortical activity [56, 90, 92–96] as well as the strongest correlation with the BOLD fMRI signal used in non-invasive neuroimaging studies [59, 68, 97–102].

We note here that different components of the recorded icEEG signal, specifically the raw-field or event-related potential (ERPs), contain additional information that could be used for the purposes of this analysis. However, ERP frequency components are more heavily influenced by lower frequency ranges, reflecting synchronized activity across larger cortical distances, which results in a poorer temporal and spatial resolution than BGA [56]. Furthermore, prior icEEG studies that have directly compared ERPs and BGA have demonstrated that a) fMRI BOLD activity tracks BGA, not ERPs [103]; b) the presence of BGA accurately predicts the presence of an ERP, and BGA magnitude is positively correlated with the size of the ERP, although the converse is not [63]; and c) BGA is more sensitive to task-dependent modulations in local cortical activity than ERPs, and BGA can distinguish between increases and decreases in neural activity (which ERPs cannot do) [63, 69, 104–106].

An important feature of ECoG recordings is the time resolution that these data provide. Time series representations of mean percent change in BGA (across trial) were calculated by comparing post-stimulus BGA power to a mean pre-stimulus baseline activity (-700 to -200 ms) (Fig 1c) [60, 79]. For each category, trials with noise or artifacts during either the baseline or post-stimulus window were discarded, resulting in a mean (+/- sd) of 46 (18) face trials; 31 (9) animate trials; 29 (8) tool trials; 49 (6) place trials; and 38 (11) word trials used in the analyses.

We also sought to characterize category-selectivity onsets per SDE per individual. We note here that the millisecond temporal resolution afforded by ECoG allows for precise latency estimates [107]. Using the BGA time-series to perform paired two-way t-tests at each time point, selectivity onset latencies were determined as the first time point at which a significant contrast (p < 0.05; corrected using the false discovery rate—FDR—procedure for multiple comparisons [108]) for a single category (against all other categories) was observed, which then remained significant for successive data points (>100 ms).

### Quantifying category-selectivity and relationship to cortical topology

To quantify category selective responses in each SDE, the *d’* (d-prime) sensitivity index was computed for each category per electrode (a total of 5 *d’* indices per electrode). The *d*’ index is an established metric in signal detection used to determine how well a target can be discriminated from competing stimuli [59, 106, 109–114]. For each category at each electrode, the mean BGA in the 100-400ms interval after stimulus onset was standardized by across trial standard deviation [59, 113]. The *d’* index was calculated as the difference between the standardized BGA for each category against all other categories:

$$d\prime =\frac{u_{j}-\frac{1}{N}\sum_{i}^{N}u_{i}}{\sqrt{\frac{1}{2}\left(o_{j}^{2}+\frac{1}{N}\sum_{i}^{N}o_{i}^{2}\right)}};i\neq j$$

where *u*_j_ is the mean response to the current category *j*; *o*_j_ is across-trial standard deviation of BGA activity to category *j*; and *u*_i_ and *o*_i_ denote the same for the other categories. Because 5 categories in all were evaluated, for each category *j*, *N* will be equal to 4. In this fashion, each electrode could be judged selective for multiple categories [112].

Significance thresholds were determined through permutation testing. For each electrode per subject, a null distribution was generated by randomly shuffling category labels across all trials and recomputing the *d’* index 10,000 times. The *p*-value for each category per electrode was determined as the fraction of shuffled *d’* indices that were greater than the actual *d’* index [113]. At the group-level, individual *p*-values were corrected for multiple comparisons (across categories and SDEs, per region and hemisphere) to an adjusted alpha (*q*) level of 0.01. Corrections for multiple comparisons were performed using the false-detection rate (FDR) procedure [108].

To test for lateral-to-medial and ventral-to-dorsal functional gradients in the VTC and LOC respectively, linear mixed effects (LME) models were generated to quantify the relationship between category-selectivity (determined by the *d*’ index) and the cortical topology while controlling for individual subject effects. For each category, SDE coordinates (in group, i.e. Talairach, space following surface-based normalization) were modeled as a fixed effect, and patient ID modeled as a random effect to control for inter-subject variability as well as non-independence (e.g. one subject contributing multiple SDEs) [lme4 and lmerTest packages in R] [115–119]. To control for spatial multicollinearity, SDE coordinates per hemisphere in each region (VTC and LOC) were mean-centered prior to inclusion in the LME models. LME models were then fitted per category for each hemisphere in each region.

Finally, to visually evaluate the spatial organization of SDE category-selectivity relative to anatomical landmarks (the MFS and LOS), SDEs with significant *d’* indices (FDR corrected *q* ≤ 0.01) for each category were visualized on the MNI N27 cortical surface (aligned to Talairach space), and color-coded by category-preference.

## Results

ECoG recordings of broadband gamma activity (BGA; 60 -120Hz) from 26 subjects (LH *n* = 16; RH *n* = 10) were analyzed to evaluate the relationship between category-selectivity and cortical topology in higher-level visual cortex. In total, 242 SDEs were evaluated (Fig 2): 159 SDEs were localized to ventral temporal cortex (VTC: LH *n* = 94, median = 5 SDEs/subject, interquartile range, IQR = 3–8.25; RH *n* = 64, median = 4.5 SDEs/subject, IQR = 4–5), and 83 SDEs were localized to lateral occipital cortex (LOC: LH *n* = 48, median = 3.5 SDEs/subject, IQR = 1.5–7; RH *n* = 35, median = 7 SDEs/subject, IQR = 3–10).

**Fig 3:**
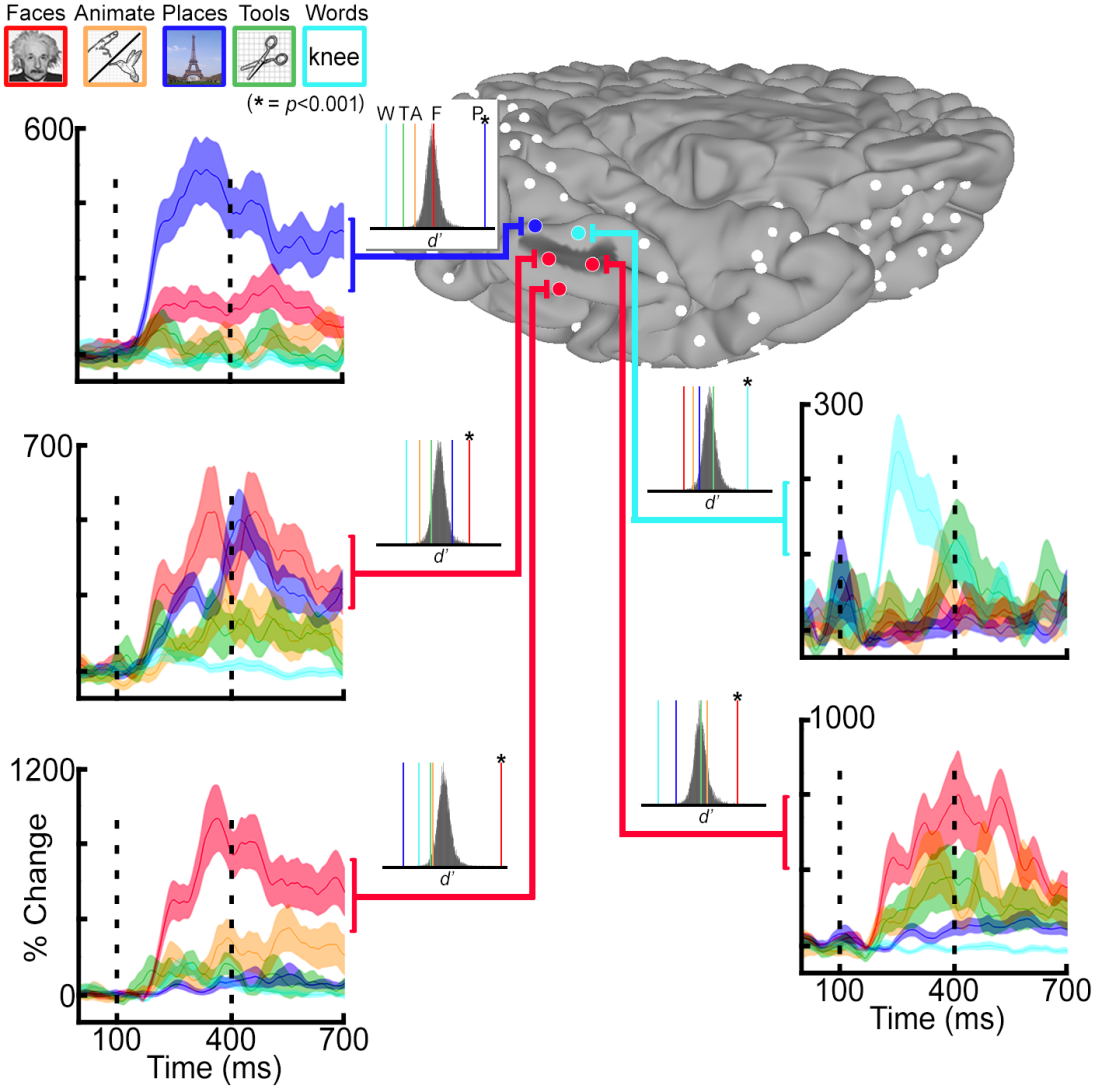
Single subject analysis for category selectivity. Single subject category-selectivity determined using the *d’* sensitivity index. Five subdural electrodes (SDEs) were localized in this individual to the vicinity of the mid-fusiform sulcus (MFS, dark gray shading on cortical surface). Time-series representations of broadband gamma activity (BGA, 60–120 Hz) for face (red), animate (orange), place (blue), tool (green), and word (cyan) stimuli are depicted for each SDE. Vertical dashed lines denote the time window for *d*’ analysis (100 to 400 ms after stimulus onset). *p*-values per category and per SDE were determined against a null distribution (insets; *n* = 10,000 permutations). Colored vertical lines denote actual *d’* index per category (colors matched to tasks, asterisks denote *p*≤0.001). In this subject, all face-selective SDEs (*n* = 3; red spheres) are localized at or lateral to the MFS, while place and word selective SDEs are localized postero-medially and antero-medially, respectively.

**Fig 4:**
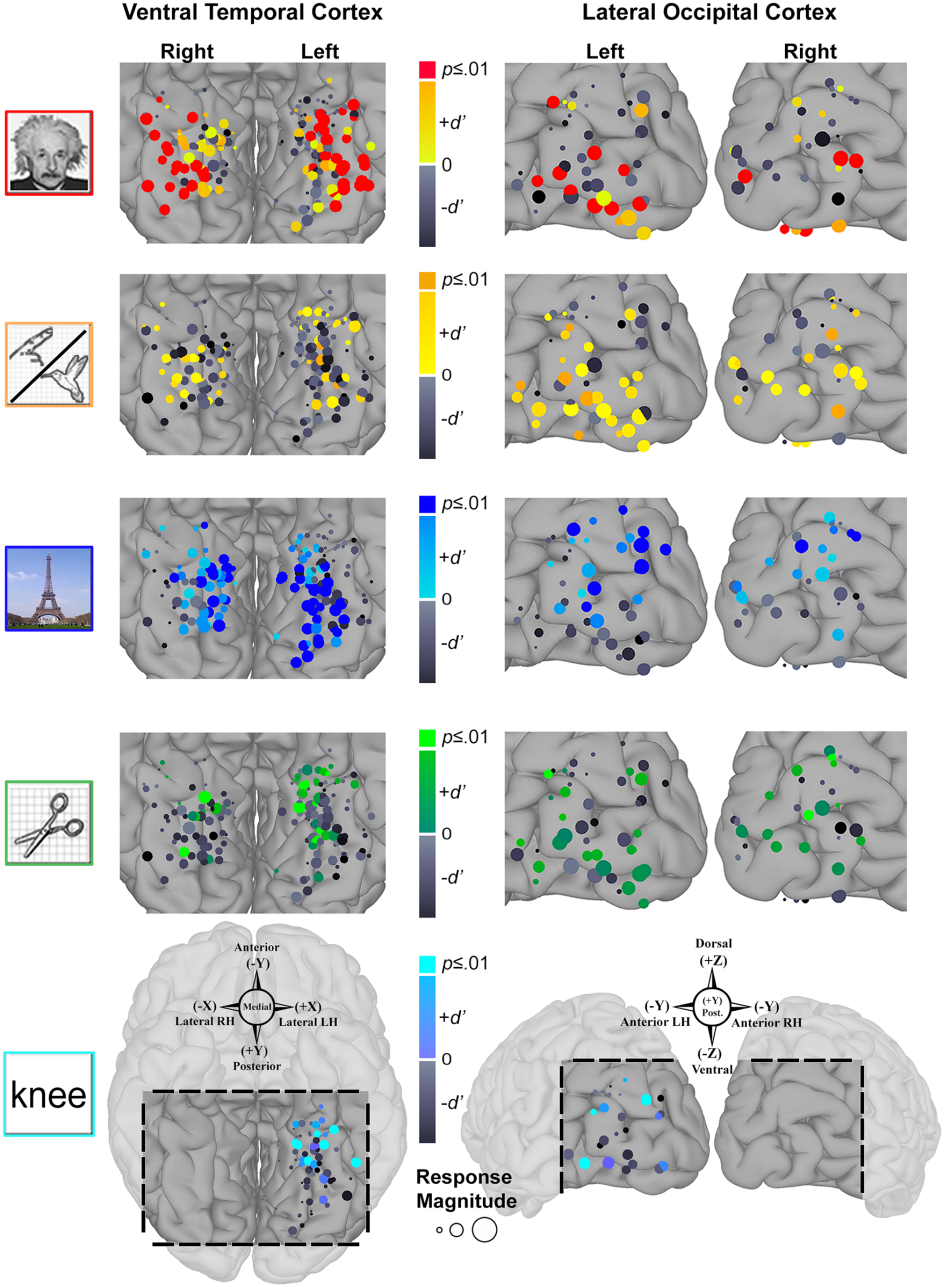
Grouped SDE and *d*’ visualization. Responsivity and preference to each category for all subdural electrodes (SDEs) over ventral temporal cortex (VTC, *right*) and lateral occipital cortex (LOC, *left*), grouped across all 26 subjects (following surface-based normalization) and visualized on the MNI N27 template brain. Compass points denote SDE coordinates (Talairach space) and direction. SDE diameter reflects normalized BGA magnitude for each category (mean BGA divided by standard deviation), scaled by the largest normalized response across categories per region (VTC and LOC are scaled differently). SDE colors reflect their d’ values per category. Positive, significant *d’* indices (FDR corrected *q* ≤ 0.01) are represented by the category-specific color-code at the top of the color bar (e.g. SDEs with significant face *d’* colored red). Positive, non-significant *d’* indices are represented as intermediate color-scales specific for each category. Negative *d’* indices are represented by gray color-scale (darker = more negative values).

At the individual level, task-dependent increases in BGA peaked at ~350—400ms after stimulus onset (Fig 3). Category-selective BGA responses (significant *d’* index at an FDR corrected *q* ≤ 0.01), organized with respect to the cortical topology, were consistently seen at the single subject level. However, the sparse sampling in each individual case precluded a comprehensive evaluation of these relationships at the single subject level, and surface-based normalization was performed to transform all SDE coordinates across subjects to a common brain space (Fig 4).

Of the 242 SDEs used in the analysis (VTC and LOC bilaterally), a total of 142 SDEs (~59%) had a significant *d*’ index for at least one category (FDR corrected *q* ≤ 0.01). In the VTC, a total of 69/94 SDEs (~73%) in the left hemisphere and 34/64 SDEs (~53%) in the right hemisphere had a significant *d’* index (FDR corrected *q* ≤ 0.01) for at least one category (Fig 4, left). In the LOC, a total of 26/48 SDEs (~54%) in the left hemisphere and 13/35 SDEs (~37%) in the right hemisphere had a significant *d’* index for at least one category (Fig 4, right). Notably, only 7 SDEs (VTC *n* = 6; LOC *n* = 1) had a significant *d’* index for a second category (both faces and places), all of which were localized in the left hemisphere to the respective sulci of interest (MFS or LOS).

### Linear mixed effects (LME) analysis of *d*’ indices with SDE coordinates

To robustly quantify the relationships between *d’* index and SDE coordinates (mm, in Talairach space), while controlling for non-independence of data within individuals, linear mixed effects (LME) models were generated for each stimulus category. We note that in the VTC, x and z coordinates were highly correlated (RH: r_s,62_ = .97, *p* = 2.2e-16; LH: *r*_s,*92*_ = -.83, *p* = 2.2e-16). Therefore only the x and y coordinates were evaluated for the VTC (z coordinate was removed). Similarly, in the LOC, the x and y coordinates were highly correlated (LH: *r*_s,*46*_ = -.94, *p* = 2.2e-16; RH: r_s,33_ = .865, *p* = 1.8e-14). Therefore only the y and z coordinates were evaluated in the LOC (x coordinate was removed). The exclusion of the z and x coordinates as predictors for VTC and LOC category selectivity, respectively, remains consistent with the anatomical principles governing structure-function hypotheses currently being tested (e.g. animacy maps in the VTC are a function of a lateral-to-medial axis).

**Fig 5:**
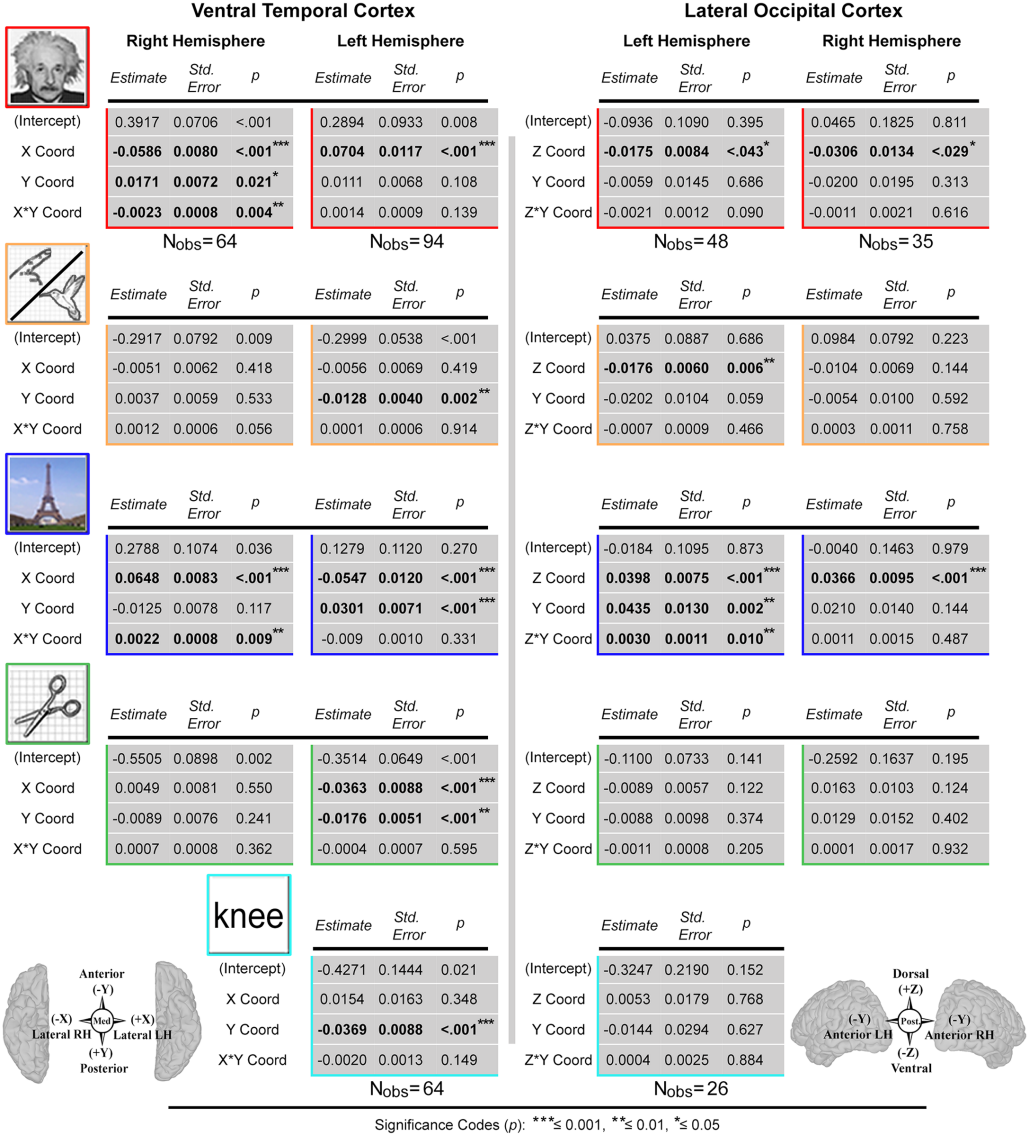
Linear mixed effects model results. Linear mixed effects (LME) models computed to quantify relationship between *d’* sensitivity index (category-selectivity) and subdural electrode (SDE) coordinates (cortical topology) for each category per hemisphere in the ventral temporal cortex (VTC; left) and lateral occipital cortex (LOC; right). Tables provide coefficient estimates, standard errors, significance levels and number of observations (N_obs_) for fixed effects predictors in each hemisphere per region. For face, animate, place, and tool LME models, the number of observations is consistent for each region and hemisphere, and thus listed once (under model results for faces). In the LOC, the fixed effects were: Z-Coord, Y-Coord, and Z*Y-Coord. In the VTC: X-Coord, Y-Coord, and X*Y-Coord. All SDE coordinates (in mm, aligned to Talairach space using surface-based normalization) were mean-centered prior to being entered into the models. Bold text denotes significant predictors, with significance levels denoted by the asterisks (legend at bottom).

In the VTC, the x and y coordinates, and the interaction term (x*y), were entered as fixed effects into the models. In the LOC, the fixed effects were entered as the y and z coordinates, and the interaction term (z*y). Given that multiple SDEs could be contributed from each individual, all models included random-effect variable intercepts for subjects to control for inter-subject variability. Complete model results for the VTC and LOC are provided in Fig 5. For brevity, only significant LME results are discussed in the following section. Scatterplots depicting univariate relationships between grouped *d*’ indices and the spatially normalized SDE coordinates of interest (Talairach space) are available in the supporting information (S1 Fig) [ggplot2 and stats packages in R] [120, 121].

#### LME analysis: ventral temporal cortex

In the right VTC, LME analysis was performed for 4 stimulus categories (faces, animate, places, and tools) using 64 SDEs (Fig 5). For face stimuli, a negative relationship was found with increasing *d’* index in the x-axis (*B* = -0.0586, *S*.*E*. = 0.0080, *p* = 6.5e-10; indicating selectivity increases laterally), a significant positive relationship with increasing selectivity in the y-axis (*B* = 0.0171, *S*.*E*. = 0.0072, *p* = .021; posteriorly), and a significant negative relationship between face-selectivity and the x*y interaction term (*B* = -0.0023, *S*.*E*. = 0.0008, *p* = 4.3e-03). For place stimuli, we found a significant positive relationship with increasing selectivity in the x-axis (*B* = 0.0648, *S*.*E*. = 0.0083, *p* = 1.2e-10; medially), and a significant positive relationship between selectivity and the x*y interaction term (*B* = 0.0022, *S*.*E*. = 0.0008, *p* = 9.2e-03). No significant associations were noted for tool- or animate-selectivity.

In the left hemisphere VTC, LME analysis was performed for 4 stimulus categories (faces, animate, places, tools) using 94 SDEs, and for 1 stimulus category (words) using 64 SDEs. For face stimuli, we found a significant positive relationship with an increasing *d’* index in the x-axis (*B* = 0.0704, *S*.*E*. = 0.0117, *p* = 3.32e-08; selectivity increases laterally). For animate stimuli, a negative relationship was observed for increasing selectivity in the y-axis (*B* = -0.0128, *S*.*E*. = 0.0040, *p* = 2.15e-03; anteriorly). For places, we found a negative relationship with increasing place-selectivity in the x-axis (*B* = -0.0547, *S*.*E*. = 0.0120, *p* = 1.53e-05; medially), and a positive relationship with increasing selectivity in the y-axis (*B* = 0.0301, *S*.*E*. = 0.0071, *p* = 5.91e-05; posteriorly). For tools, we found a negative relationship with increasing selectivity in the x-axis (*B* = -0.0363, *S*.*E*. = 0.0088, *p* = 9.00e-05; medially), and a negative relationship with the y-axis (*B* = -0.0176, *S*.*E*. = 0.0051, *p* = 9.28e-04; anteriorly). For words, a negative relationship was observed with increasing selectivity in the y-axis (*B* = -0.0369, *S*.*E*. = 0.0088, *p* = 9.67e-05; anteriorly).

#### LME analysis: lateral occipital cortex

In the left LOC, LME analysis was performed for 4 stimulus categories (faces, animate, places, and tools) using 48 SDEs and for 1 stimulus category (words) using 26 SDEs (Fig 5). For both face and animate stimuli, we found significant negative relationships with increasing *d’* indices in the z-axis (face *B* = -0.0175, *S*.*E*. = 0.0084, *p* = 0.043; animate *B* = -0.0176, *S*.*E*. = 0.0060, *p* = 5.6e-03; selectivity increases ventrally for both). For places, we found a significant positive relationship with increasing selectivity in the z-axis (*B* = 0.0398, *S*.*E*. = 0.0075, *p* = 3.8e-06; dorsally), a significant positive relationship with the y-axis (*B* = 0.0435, *S*.*E*. = 0.0130, *p* = 1.7e-03; anteriorly), as well as a significant positive relationship with the y*z interaction term (*B* = 0.0030, *S*.*E*. = 0.0011, *p* = 9.8e-03). No significant associations were noted for tool or word-selectivity.

Finally, in the right LOC, LME analysis was performed for 4 stimulus categories (faces, places, tools, and animate) using 35 SDEs. For faces, we found a significant negative relationship with increasing selectivity in the z-axis (*B* = -0.0306, *S*.*E*. = 0.0134, *p* = .029; selectivity increases ventrally), and for places we found a significant positive relationship with increasing selectivity in the z-axis (*B* = 0.0366, *S*.*E*. = 0.0095, *p* = 6.0e-04; dorsally). No significant associations were noted for tool- or animate-selectivity.

### Topology of category-selectivity

**Fig 6:**
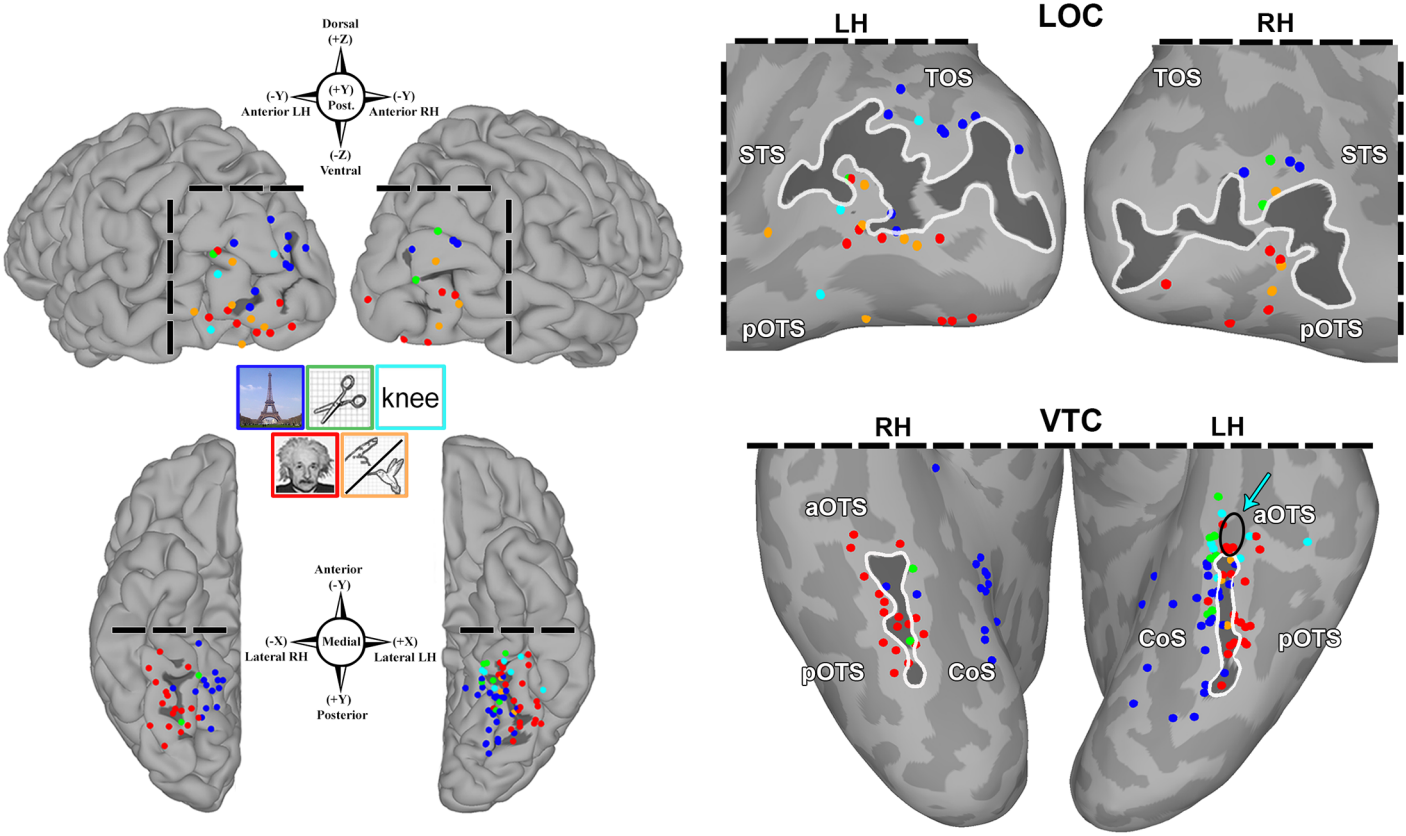
Spatial organization of category-selectivity. All subdural electrodes (SDEs) with significant category-selectivity (FDR corrected *q*≤0.01) are visualized on the MNI N27 template brain (aligned to Talairach coordinate space) after surface based normalization. SDEs are color-coded by the category of preference (matched to image legends). Compass points denote SDE coordinates (Talairach space) and direction. Left: Pial surface maps of lateral occipital cortex (LOC, *top*) and ventral temporal cortex (VTC, *bottom*). Right: inflated surfaces for these regions with the lateral-occipital sulcus (LOS) and mid-fusiform sulcus (MFS) delineated by dark gray shades and white contours, and adjacent sulci delineated by lighter gray shades (TOS, transverse occipital sulcus; STS, superior temporal sulcus; p/aOTS, posterior/anterior occipito-temporal sulcus; CoS, collateral sulcus). In the LOC, all 13 face-selective (red; LH *n* = 8; RH *n* = 5) and 9 animate-selective (orange; LH *n* = 6; RH *n* = 3) SDEs are localized at or below the LOS, while all 12 place- (blue; LH *n* = 9; RH *n* = 3) and 3 tool-selective (green; LH *n* = 1; RH *n* = 2) SDEs are localized at or dorsal to the LOS. In the VTC, all 3 animate- (LH only) and 42/45 face-selective (LH *n* = 27/28; RH *n* = 15/17) SDEs are localized to-or-lateral to the MFS, while all 43 place- (LH *n* = 29; RH *n* = 14) and 9 tool-selective (LH *n* = 7; RH *n* = 2) SDEs are localized to-or-medial to the MFS. All 6 word-selective (cyan, LH only) SDEs are localized anteriorly at the intersection of the MFS and aOTS (arrow and black circle), interspersed with animate, tool, and anteriorly-localized face-selective SDEs.

To evaluate the spatial relationship of category-selective SDEs with respect to cortical folding patterns, all SDEs with significant *d’* indices were visualized on the MNI N27 brain surface (in Talairach space), and color-coded by category preference (Fig 6). Notably, all animate-selective (LH *n* = 3/3) and nearly all face-selective (LH *n* = 27/28; RH *n* = 15/17) SDEs were localized to or lateral to the mid-fusiform sulcus (MFS) in the VTC bilaterally. Similarly, all place-selective (LH *n* = 29/29; RH *n* = 14/14) and tool-selective SDEs (LH *n* = 7/7; RH *n* = 2/2) were localized to or medial to the MFS bilaterally. Additionally both tool-selective and word-selective (LH *n* = 6/6) SDEs were clustered along the anterior boundary of the mid-fusiform sulcus in the left VTC. In addition to tools, word-selective SDEs were also interspersed with anteriorly localized face- and animate-selective SDEs.

In the LOC, bilaterally, a similar arrangement of category-selectivity with respect to the lateral occipital sulcus (LOS) was observed. All face-selective (LH *n* = 8/8; RH *n* = 5/5) and animate-selective (LH 6/6; RH *n* = 3/3) SDEs were uniformly localized at or inferior to the LOS, while all place-selective (LH *n* = 9/9; RH *n* = 3/3) and tool-selective (LH *n* = 1/1; RH *n* = 2/2) SDEs were localized at or superior to the LOS. However, no discernable spatial arrangement of word-selective (LH *n* = 3) SDEs could be observed.

We also examined the spatial organization of the 105 remaining SDEs without significant *d*’ indices (S3 Fig). Of these, 34 SDEs (LH *n* = 17; RH *n* = 17) demonstrated little to any change in BGA (i.e. were non-responsive), and all were localized at the boundaries of the VTC/LOC: at-or-anterior to the MFS in the VTC (LH *n* = 14; RH *n* = 12) and at-or-superior to the transverse occipital sulcus in the LOC (LH *n* = 3; RH *n* = 5). The remaining 71 SDEs were largely interspersed amongst category-selective SDEs, with one notable exception: in the VTC bilaterally, no non-selective SDEs were localized postero-lateral to the MFS, in regions of face-selectivity. In the remaining regions, d’ index values of non-selective SDEs were largely congruent with *d*’ indices of the surrounding category-selective SDEs, consistent with the gradients of category-selectivity observed in the LME analyses and scatterplots (S1 Fig).

Finally, for VTC and LOC category-selective SDEs, we investigated the timing of selectivity emergence for each category (S2 Fig). Bilaterally, median selectivity onset latencies were between ~150 to ~250 ms overall. In the left hemisphere LOC, median (sd) onset latencies were: 133 ms for (n = 1) tool-selective SDEs; 138 (39) ms for (n = 6) animate-selective SDEs; 155 (35) ms for (n = 8) face-selective SDEs; 194 (54) ms for (n = 3) word-selective SDEs; and 219 (43) ms for (n = 9) place-selective SDEs. In the left VTC, median (sd) onset latencies were: 153 (55) ms for (n = 6) word-selective SDEs; 162 (50) ms for (n = 28) face-selective SDEs; 172 (61) ms for (n = 29) place-selective SDEs; 193 (36) ms for (n = 7) tool-selective SDEs; and 250 (52) ms for (n = 3) animate-selective SDEs.

In the right LOC, median (sd) onset latencies were: 158 (42) ms for (n = 5) face-selective SDEs; 191 (84) ms for (n = 2) tool-selective SDEs; 238 (81) ms for (n = 3) place-selective SDEs; and 305 (101) ms for (n = 3) animate-selective SDEs. In the right VTC, median (sd) onset latencies were: 163 (88) ms for (n = 2) tool-selective SDEs; 173 (35) ms for (n = 14) place-selective SDEs; and 203 (74) ms for (n = 17) face-selective SDEs.

Systematic comparisons of onset latencies of category-selectivity (S3 Fig), within regions per hemisphere (Wilcoxon signrank test, FDR corrected for multiple comparisons), revealed no significant differences across categories in any of the regions (q > 0.05). We note that in the LOC, bilaterally, face-selectivity onset appeared to trend earlier than place-selectivity onset. However, variable sample-sizes of these category-selective SDEs, both within and across the VTC/LOC bilaterally limit the power of these comparisons.

## Discussion

We utilized a surface-based grouped icEEG analyses, combined across a large cohort (*n* = 26; LH *n* = 16, RH *n* = 10), to provide a population-level electrophysiological evaluation of the topology of category-selectivity in higher-order visual cortex. We demonstrate a consistent spatial organization of category-selective regions with respect to specific anatomical landmarks in the ventral temporal and lateral occipital cortical complexes (VTC and LOC). Importantly, our findings advance prior work by demonstrating that the use of surface-based normalization strategies in grouped icEEG analyses preserves structure-function coupling in a common brain space. In doing so, we provide a method to circumvent the sparse-sampling problem that has constrained the broader application of icEEG to the study of cognitive function at the single subject level [60, 122].

### Structure-function coupling in higher-level visual cortex

Our data reveal significant associations between category-selectivity with both lateral-to-medial and posterior-to-anterior axes in the VTC, as well as a dorsal-to-ventral axis in LOC, bilaterally.

In the LOC, the lateral-occipital sulcus (LOS) provides a consistent boundary for transitions in the selectivity between living (face and animate) and non-living (place and tool) stimuli: face and animate selective regions are constrained at or ventral to the LOS, while place and tool selective regions are constrained dorsally. Notably, face- and animate selective SDEs are interspersed on the ventral aspects of the LOC in a fashion consistent with prior fMRI studies that demonstrate alternating regions of face and limb-selectivity [21, 123, 124]. Additionally, in the left LOC, tool stimuli elicit strong, but non-selective activations in SDEs localized ventral to the LOS. Although the ventral LOC exhibits an overall greater selectivity for living stimulus categories, the role of the LOC in more general visual form processing is well documented [18, 21, 37, 125, 126].

In the VTC, the mid-fusiform sulcus (MFS) provides a consistent boundary for transitions in the selectivity between living (face and animate) and non-living (place and tool) stimuli: face and animate selective areas are constrained at or lateral to the MFS, while place and tool selective regions are constrained at or medial to the MFS. Furthermore, in the left VTC, the anterior aspect of the MFS predicts the location of word, animate, and tool selective responses, suggesting that the VTC may possess additional functional gradients along the postero-anterior anatomical axis. Notably, regions demonstrating word-selectivity are clustered around the intersection of the occipito-temporal sulcus (OTS) and the anterior MFS (Fig 6), consistent with prior studies of word selectivity that have localized cortical regions sensitive to orthographic stimuli to the general vicinity of the OTS (i.e. the visual word-form area) [13, 17, 45, 76–78, 112, 127]. Given that word, tool, animate, and face stimuli are typically dependent on central vision [23, 43, 44, 46], the co-localization of word-selectivity with these other categories remains consistent with our original prediction that word-selectivity should be observed in regions with foveal bias. Taken together, these findings support the hypothesis that distinct functional maps (e.g. animacy and eccentricity bias) are arranged along similar organization principles within the same expanse of cortical tissue [23].

While the locations of VTC and LOC category-selectivity reported here are consistent with an extensive body of invasive and non-invasive neuroimaging studies [9, 14, 19, 20, 37, 38, 50, 61, 74, 105, 128–138], our findings are novel in that they provide direct electrophysiological support for hypotheses of hierarchical information structuring using icEEG data *combined across many individuals*. Such hypotheses propose that small-scale functional representations are nested together within larger-scale functional maps in higher-level visual cortex, facilitating object categorization by the visual system (and possibly other higher-order cognitive systems) by enabling the extraction of different levels of categorical detail at different spatial scales (i.e. small scale for face information, larger scale for animacy information) [22, 23]. This hierarchical information structure is believed to arise from the distinct anatomical organization of these regions, as the MFS and LOS also predict transitions in cortical micro- and macro-architecture (e.g. cyto- and receptor architectonics and white-matter structural networks, respectively) [25, 33, 34, 36]. Such organization may speed visual categorization by directing unrelated visual information to distinct neural networks operating in parallel (e.g. details pertaining to scenes vs. faces), while related visual information (e.g. faces and body-parts) converge onto shared neural substrates [23, 139].

Notably, such a parallel network organization is independently supported by the result from our BGA time-series analyses, which revealed no significant regional differences in the emergence of category-selectivity across conditions (between ~150 and ~250 ms). The relative similarities in selectivity onset latencies both across categories within the VTC, as well as between the VTC and LOC, support the hypothesis that visual information is received and processed in these regions in a largely independent fashion [21, 25, 113, 140, 141]. Although the small and unequal numbers of category-selective SDEs (within and across regions) makes a definitive assessment of our time-series results impossible, we note that the latencies of selectivity reported here are consistent with prior intracranial work in both non-human primates and humans [1, 3, 61, 63, 64, 66, 68, 128, 130, 142, 143].

To date, evidence for hierarchical coding models has come almost exclusively from non-invasive neuroimaging studies. Although a recent electrophysiological study has also reported large-scale animacy distinctions along the MFS [59], the analysis in this study was restricted to a small sample size (*n* = 6; LH 3, RH 3) and constrained to the individual level. Our work here validates their findings in a larger population, extends the investigation to the LOC, and broadens the stimulus classification to include tools and words. The high spatial resolution of icEEG enabled us to confirm the boundaries of these higher-level visual regions via the consistent localization of *non-responsive* SDEs anterior to the MFS in the VTC and superior to the transverse occipital sulcus in the LOC. The finding that no *non-selective* SDEs are localized within postero-lateral VTC, bilaterally, supports prior reports on the highly selective nature of this region (specifically with respect to faces) [141, 144]. Notably, our observation that SDEs with dual-selectivity were localized within the MFS or LOS indicates that either our recordings average across multiple modules arranged in proximity to each other within the sulcus, or that the transitions between neuronal clusters tuned to specific categories may be a gradual one [40]. While the recording scale of the SDEs used clinically does not allow us to distinguish between these two possibilities, our results nevertheless provide novel support that these sulci—the MFS and LOS—are critical to the functional topology of higher-level visual cortex.

### Grouped icEEG: a solution to the sparse-sampling problem

The sparse-sampling problem has been a long-standing limitation of icEEG, to which the recent development of surface-based grouped techniques provides a viable and much-needed solution [56, 87, 98, 122]. In the current study, we combined data across 26 different subjects, each introducing a unique source of topological and pathological variability. The nonlinear transformation utilized here to map 242 SDEs into a common brain space preserved structure-function coupling across this heterogenous population, thus validating surface-based approaches to grouped icEEG. Furthermore, our findings also demonstrate a consistency of functional representation in our patient population—both amongst themselves and with respect to healthy subjects—thereby validating the use of patients with focal epilepsy for the study of cognitive function. In doing so, our work advances the field of icEEG by broadening its potential to contribute to the study of human cognition beyond the single subject level.

### Limitations

Three main limitations of this work are apparent to us. The first is that we include only subjects implanted with SDEs, which record from the gyral crowns, and may be biased against activity arising from sulcal sources. Notably, prior literature focusing on word-, limb-, and body-selectivity in the VTC has reported regions localized in or near the OTS [13, 21, 27, 45, 123, 145, 146]. The paucity of VTC animate selectivity reported in the current study, as well as the clustering of word-selectivity on gyral crowns in the anterior MFS, may have resulted from this gyral bias. To investigate this possibility, future icEEG work will integrate SDE data with data obtained from penetrating depth electrodes or stereotactic EEG [69].

A second limitation is the inconsistency in the low-level visual features of our stimuli (e.g. colored images for places vs. gray-scale face stimuli vs. line-drawings of tools/animate stimuli), which provide a potential confound in our analysis. However, higher-level visual regions are known to be invariant to changes in low-level visual features, and to maintain visual selectivity across a large spectrum of visual information, including color [23, 147–154]. Such invariance is reflected here by the co-localization of SDEs exhibiting category-selectivity for visually disparate stimuli along sulcal boundaries in the VTC and LOC. More specifically, while place and tool stimuli were the least similar in terms of low-level features (e.g. real color images of large, naturalistic stimuli vs. line-drawings of small, handheld objects) both were clustered together medial to the MFS. Similarly, in the LOC, face and animate stimuli (gray-scale vs. line-drawings, respectively) were clustered together ventrally with respect to the LOS. Future icEEG studies incorporating a more diverse selection of category classes will be needed to more fully substantiate our findings, as our results are limited by the use of only two animate and inanimate classes.

The third limitation is that our stimulus set does not allow us to unequivocally claim that the abstract semantic concept of “animacy” is the driving force behind the topological organization we observe. Notably, prior studies have argued that animacy distinctions in higher-order visual areas may simply be a by-product of shape similarities between stimuli of related categories (but see [155]) [156–161]. Nevertheless, category-specific functional gradients along abstract semantic boundaries (e.g. animacy) have been previously demonstrated in the congenitally blind [162]. Additionally, in a recent study describing the topographic representation of body parts in the VTC and LOC, shape similarities were found to be insufficient to explain the architecture of the body-maps observed. Specifically, the authors demonstrated that regions preferential to a specific class of body-parts (e.g. upper limbs) were more responsive to within-class images, despite their greater dissimilarities in shape (e.g. hands and elbows), than to more similarly shaped images from distinct classes (e.g. feet and knees—lower-limbs) [27]. Finally, a recent computational study has suggested how functional representations along abstract semantic boundaries (specifically animacy) could be achieved via top-down influences (reflected in supervised learning models); with their most successful models incorporating both visual and semantic information [163].

Thus, a final account of the functional topology within higher-order visual regions will likely need to account for both low-level visual features (e.g. shape) as well as influences from semantic or categorical dimensions [23, 37, 40, 51, 155, 161, 163–165]. This interpretation is in line with evidence from recent monkey electrophysiological studies suggesting that core (i.e. invariant) object recognition in primates (both human and non-human) rely on non-semantic representations of visual features, upon which semantic knowledge (in humans) can subsequently be learned [155, 159, 161, 163].

### Conclusion

We provide a grouped icEEG investigation of VTC and LOC category-selectivity, and demonstrate unequivocal evidence for structure-function coupling through direct electrophysiological recordings in a large human cohort. Our findings support hypotheses of hierarchical information structuring in higher-level visual cortex, via the generation of large-scale functional maps (e.g. animacy) from nested functional representations consequent to this structure-function coupling.

Surface-based strategies to icEEG analyses provide novel opportunities for researchers to pool ECoG datasets across centers. Given the relative rarity of icEEG data in many cortical regions of interest, the adoption of such collaborative strategies could provide an invaluable tool to greatly expand the relevant application of high spatiotemporal resolution icEEG to the study of higher-level cognitive function.

## Supporting Information

S1 Dataset Categories data for LH VTC cohort. S1 Dataset comprises a Matlab file (.mat) containing 6 variables. For the 94 VTC SDEs across the 16 subjects with left hemispheric coverage, there exists a distinct matrix for the BGA mean, BGA standard deviation, and *d*’ indices for each of the 5 categories tested. The order of the categories in these variables is identical to the order of the listed in the “Categories” cell variable. Additionally, for each SDE, a “Pt_Coords_LH_VTC” matrix contains the SDE coordinates in group space (MNI N27 brain, aligned to Talairach space). Finally, there is also a cell labeled “pt_dprime_final_shuf_LH_VTC” which contains all 10,000 shuffled *d’* indices for each subject’s SDE. The use of cell format enables the number of SDE’s each subject contributed to be seen. The number of SDEs contained in each cell corresponds to the number of VTC SDEs for the LH cohort listed in Table 1. This information can be used to identify which SDE belongs to which subject in the other matrices contained in this S1 dataset.
(MAT)

S2 Dataset Categories data for RH VTC cohort. The S2 dataset contains the identical matrices and cells as described in for the S1 dataset. In this case, these data are for the 64 VTC SDEs across the 10 subjects with right hemispheric coverage.
(MAT)

S3 Dataset Categories data for LOC cohort bilaterally. The S3 dataset contains the identical matrices and cells as described in for the S1 dataset. In this case, these data are for the 48 LH LOC and 35 RH LOC SDEs across the entire bilateral cohort of 26 subjects.
(MAT)

S1 Fig *d’* sensitivity vs. SDE coordinates. Scatterplots depict grouped *d*’ indices for each category plotted vs. subdural electrode (SDE) coordinates (in Talairach space) per hemisphere in each region. In the ventral temporal cortex (VTC; RH *n* = 64, LH *n* = 94), comparisons were made against the x and y coordinates. In the lateral occipital cortex (LOC; LH *n* = 48, RH *n* = 35), comparisons were made with the z and y coordinates. For each plot, regression lines were fitted (color-coded by category), and the strengths of association were estimated using Spearman correlations (bottom right, bold text denotes FDR corrected *q* ≤ 0.05, for multiple comparison across categories and SDEs per region and hemisphere). Spearman correlations were selected (over Pearson’s) for their robustness to outlier influence and smaller sample sizes. Furthermore, Spearman’s correlations test for monotonic relationships, and the relationships between d’ indices and SDE coordinates are not known a priori to be linear.
(TIF)

S2 Fig Onset latencies of category-selectivity. Box plots depict median onset latency of category-selectivity in the left (LH) and right (RH) hemisphere lateral occipital cortex (LOC) and ventral temporal cortex (VTC), for the five categories of interest: words (cyan), tools (green), places (blue), faces (red) and non-face animate (body-parts and animals) stimuli. Timing of the onset of selectivity was evaluated for each category-selectivity SDE per subject, and determined using pairwise comparisons in broadband gamma activity time-series, for each category against all others. No significant differences between categories (following corrections for multiple comparisons) were noted, although low sample sizes likely underpowered these contrasts. Word stimuli were not tested in the right hemisphere, and in the RH VTC, no significant animate SDEs were observed.
(TIF)

S3 Fig *d’* distribution of non-significant SDEs. Non category-selective subdural electrodes (SDEs) are visualized on the MNI N27 template brain (aligned to Talairach coordinate space) after surface based normalization. SDEs are color-coded by the category with the largest *d*’ index for that electrode (matched to image legends). SDE diameter reflects the magnitude of the *d*’ value for that category, scaled by the largest d’ value across categories per region (regions per hemisphere are scaled differently). Compass points denote SDE coordinates (Talairach space) and direction. Notably, in the postero-lateral aspects of bilateral ventral temporal cortex, no non-significant SDEs are observed.
(TIF)

## References

[1] DiCarlo JJ, Zoccolan D, Rust NC. How does the brain solve visual object recognition? Neuron. 2012;73(3):415–34. Epub 2012/02/14. 10.1016/j.neuron.2012.01.010 22325196 PMC3306444

[2] Barton JJ. Higher cortical visual deficits. Continuum. 2014;20(4 Neuro-ophthalmology):922–41. 10.1212/01.CON.0000453311.29519.67 . 25099101 PMC10563980

[3] Allison T, Ginter H, McCarthy G, Nobre AC, Puce A, Luby M, et al Face recognition in human extrastriate cortex. J Neurophysiol. 1994;71(2):821–5. . 8176446 10.1152/jn.1994.71.2.821

[4] Desimone R, Albright TD, Gross CG, Bruce C. Stimulus-selective properties of inferior temporal neurons in the macaque. J Neurosci. 1984;4(8):2051–62. . 6470767 10.1523/JNEUROSCI.04-08-02051.1984 PMC6564959

[5] Downing PE, Jiang Y, Shuman M, Kanwisher N. A cortical area selective for visual processing of the human body. Science. 2001;293(5539):2470–3. . 11577239 10.1126/science.1063414

[6] Gauthier I, Tarr MJ, Moylan J, Skudlarski P, Gore JC, Anderson AW. The fusiform "face area" is part of a network that processes faces at the individual level. J Cogn Neurosci. 2000;12(3):495–504. . 10931774 10.1162/089892900562165

[7] Gross CG, Bender DB, Rocha-Miranda CE. Visual receptive fields of neurons in inferotemporal cortex of the monkey. Science. 1969;166(3910):1303–6. . 4982685 10.1126/science.166.3910.1303

[8] Haxby JV, Grady CL, Horwitz B, Ungerleider LG, Mishkin M, Carson RE, et al Dissociation of object and spatial visual processing pathways in human extrastriate cortex. Proc Natl Acad Sci U S A. 1991;88(5):1621–5. 2000370 10.1073/pnas.88.5.1621 PMC51076

[9] Peelen MV, Downing PE. Selectivity for the human body in the fusiform gyrus. J Neurophysiol. 2005;93(1):603–8. 10.1152/jn.00513.2004 . 15295012

[10] Sergent J, Ohta S, MacDonald B. Functional neuroanatomy of face and object processing. A positron emission tomography study. Brain. 1992;115 Pt 1:15–36. . 1559150 10.1093/brain/115.1.15

[11] Tanaka K. Inferotemporal cortex and object vision. Annu Rev Neurosci. 1996;19:109–39. Epub 1996/01/01. 10.1146/annurev.ne.19.030196.000545 . 8833438

[12] Chao LL, Haxby JV, Martin A. Attribute-based neural substrates in temporal cortex for perceiving and knowing about objects. Nat Neurosci. 1999;2(10):913–9. 10.1038/13217 . 10491613

[13] Cohen L, Dehaene S, Naccache L, Lehericy S, Dehaene-Lambertz G, Henaff MA, et al The visual word form area: spatial and temporal characterization of an initial stage of reading in normal subjects and posterior split-brain patients. Brain. 2000;123 (Pt 2):291–307. Epub 2000/01/29. . 10648437 10.1093/brain/123.2.291

[14] Epstein R, Kanwisher N. A cortical representation of the local visual environment. Nature. 1998;392(6676):598–601. Epub 1998/04/29. 10.1038/33402 . 9560155

[15] Haxby JV, Hoffman EA, Gobbini MI. The distributed human neural system for face perception. Trends Cogn Sci. 2000;4(6):223–33. . 10827445 10.1016/s1364-6613(00)01482-0

[16] Kanwisher N, McDermott J, Chun MM. The fusiform face area: a module in human extrastriate cortex specialized for face perception. J Neurosci. 1997;17(11):4302–11. Epub 1997/06/01. . 9151747 10.1523/JNEUROSCI.17-11-04302.1997 PMC6573547

[17] Nobre AC, Allison T, McCarthy G. Word recognition in the human inferior temporal lobe. Nature. 1994;372(6503):260–3. 10.1038/372260a0 . 7969469

[18] Malach R, Reppas JB, Benson RR, Kwong KK, Jiang H, Kennedy WA, et al Object-related activity revealed by functional magnetic resonance imaging in human occipital cortex. Proc Natl Acad Sci U S A. 1995;92(18):8135–9. 7667258 10.1073/pnas.92.18.8135 PMC41110

[19] Mahon BZ, Caramazza A. Concepts and categories: a cognitive neuropsychological perspective. Annu Rev Psychol. 2009;60:27–51. 10.1146/annurev.psych.60.110707.163532 18767921 PMC2908258

[20] Op de Beeck HP, Haushofer J, Kanwisher NG. Interpreting fMRI data: maps, modules and dimensions. Nat Rev Neurosci. 2008;9(2):123–35. 10.1038/nrn2314 18200027 PMC2731480

[21] Weiner KS, Grill-Spector K. Neural representations of faces and limbs neighbor in human high-level visual cortex: evidence for a new organization principle. Psychol Res. 2013;77(1):74–97. 10.1007/s00426-011-0392-x 22139022 PMC3535411

[22] Brants M, Baeck A, Wagemans J, de Beeck HP. Multiple scales of organization for object selectivity in ventral visual cortex. Neuroimage. 2011;56(3):1372–81. 10.1016/j.neuroimage.2011.02.079 . 21376816

[23] Grill-Spector K, Weiner KS. The functional architecture of the ventral temporal cortex and its role in categorization. Nat Rev Neurosci. 2014;15(8):536–48. 10.1038/nrn3747 24962370 PMC4143420

[24] Dilks DD, Julian JB, Paunov AM, Kanwisher N. The occipital place area is causally and selectively involved in scene perception. J Neurosci. 2013;33(4):1331–6a. 10.1523/JNEUROSCI.4081-12.2013 23345209 PMC3711611

[25] Gomez J, Pestilli F, Witthoft N, Golarai G, Liberman A, Poltoratski S, et al Functionally defined white matter reveals segregated pathways in human ventral temporal cortex associated with category-specific processing. Neuron. 2015;85(1):216–27. 10.1016/j.neuron.2014.12.027 . 25569351 PMC4287959

[26] Kujovic M, Zilles K, Malikovic A, Schleicher A, Mohlberg H, Rottschy C, et al Cytoarchitectonic mapping of the human dorsal extrastriate cortex. Brain Struct Funct. 2013;218(1):157–72. 10.1007/s00429-012-0390-9 22354469 PMC3535362

[27] Orlov T, Makin TR, Zohary E. Topographic representation of the human body in the occipitotemporal cortex. Neuron. 2010;68(3):586–600. 10.1016/j.neuron.2010.09.032 . 21040856

[28] Pyles JA, Verstynen TD, Schneider W, Tarr MJ. Explicating the face perception network with white matter connectivity. PLoS One. 2013;8(4):e61611 10.1371/journal.pone.0061611 23630602 PMC3632522

[29] Saygin ZM, Osher DE, Koldewyn K, Reynolds G, Gabrieli JD, Saxe RR. Anatomical connectivity patterns predict face selectivity in the fusiform gyrus. Nat Neurosci. 2012;15(2):321–7. 10.1038/nn.3001 22197830 PMC3267901

[30] Yeatman JD, Weiner KS, Pestilli F, Rokem A, Mezer A, Wandell BA. The vertical occipital fasciculus: A century of controversy resolved by in vivo measurements. Proc Natl Acad Sci U S A. 2014;111(48):E5214–23. 10.1073/pnas.1418503111 25404310 PMC4260539

[31] Martin A, Wiggs CL, Ungerleider LG, Haxby JV. Neural correlates of category-specific knowledge. Nature. 1996;379(6566):649–52. 10.1038/379649a0 . 8628399

[32] Levy I, Hasson U, Avidan G, Hendler T, Malach R. Center-periphery organization of human object areas. Nat Neurosci. 2001;4(5):533–9. 10.1038/87490 . 11319563

[33] Weiner KS, Golarai G, Caspers J, Chuapoco MR, Mohlberg H, Zilles K, et al The mid-fusiform sulcus: a landmark identifying both cytoarchitectonic and functional divisions of human ventral temporal cortex. Neuroimage. 2014;84:453–65. 10.1016/j.neuroimage.2013.08.068 24021838 PMC3962787

[34] Caspers J, Palomero-Gallagher N, Caspers S, Schleicher A, Amunts K, Zilles K. Receptor architecture of visual areas in the face and word-form recognition region of the posterior fusiform gyrus. Brain Struct Funct. 2015;220(1):205–19. 10.1007/s00429-013-0646-z . 24126835

[35] Caspers J, Zilles K, Eickhoff SB, Schleicher A, Mohlberg H, Amunts K. Cytoarchitectonical analysis and probabilistic mapping of two extrastriate areas of the human posterior fusiform gyrus. Brain Struct Funct. 2013;218(2):511–26. 10.1007/s00429-012-0411-8 22488096 PMC3580145

[36] Lorenz S, Weiner KS, Caspers J, Mohlberg H, Schleicher A, Bludau S, et al Two New Cytoarchitectonic Areas on the Human Mid-Fusiform Gyrus. Cereb Cortex. 2015 10.1093/cercor/bhv225 . 26464475 PMC6248695

[37] Konkle T, Caramazza A. Tripartite organization of the ventral stream by animacy and object size. J Neurosci. 2013;33(25):10235–42. 10.1523/JNEUROSCI.0983-13.2013 23785139 PMC3755177

[38] Hasson U, Harel M, Levy I, Malach R. Large-scale mirror-symmetry organization of human occipito-temporal object areas. Neuron. 2003;37(6):1027–41. . 12670430 10.1016/s0896-6273(03)00144-2

[39] Nasr S, Liu N, Devaney KJ, Yue X, Rajimehr R, Ungerleider LG, et al Scene-selective cortical regions in human and nonhuman primates. J Neurosci. 2011;31(39):13771–85. Epub 2011/10/01. 10.1523/JNEUROSCI.2792-11.2011 . 21957240 PMC3489186

[40] Sha L, Haxby JV, Abdi H, Guntupalli JS, Oosterhof NN, Halchenko YO, et al The animacy continuum in the human ventral vision pathway. J Cogn Neurosci. 2015;27(4):665–78. 10.1162/jocn_a_00733 . 25269114

[41] Konkle T, Oliva A. A real-world size organization of object responses in occipitotemporal cortex. Neuron. 2012;74(6):1114–24. 10.1016/j.neuron.2012.04.036 22726840 PMC3391318

[42] Kriegeskorte N, Mur M, Ruff DA, Kiani R, Bodurka J, Esteky H, et al Matching categorical object representations in inferior temporal cortex of man and monkey. Neuron. 2008;60(6):1126–41. 10.1016/j.neuron.2008.10.043 19109916 PMC3143574

[43] Hasson U, Levy I, Behrmann M, Hendler T, Malach R. Eccentricity bias as an organizing principle for human high-order object areas. Neuron. 2002;34(3):479–90. . 11988177 10.1016/s0896-6273(02)00662-1

[44] Dehaene S. Reading in the Brain: The New Science of How We Read: Penguin; 2009.

[45] Dehaene S, Cohen L. The unique role of the visual word form area in reading. Trends Cogn Sci. 2011;15(6):254–62. Epub 2011/05/20. S1364-6613(11)00073-8 [pii] 10.1016/j.tics.2011.04.003 . 21592844

[46] Malach R, Levy I, Hasson U. The topography of high-order human object areas. Trends Cogn Sci. 2002;6(4):176–84. . 11912041 10.1016/s1364-6613(02)01870-3

[47] Jerbi K, Ossandon T, Hamame CM, Senova S, Dalal SS, Jung J, et al Task-related gamma-band dynamics from an intracerebral perspective: review and implications for surface EEG and MEG. Hum Brain Mapp. 2009;30(6):1758–71. Epub 2009/04/04. 10.1002/hbm.20750 . 19343801 PMC6870589

[48] Lachaux JP, Rudrauf D, Kahane P. Intracranial EEG and human brain mapping. J Physiol Paris. 2003;97(4–6):613–28. Epub 2004/07/10. 10.1016/j.jphysparis.2004.01.018 . 15242670

[49] Friston KJ, Rotshtein P, Geng JJ, Sterzer P, Henson RN. A critique of functional localisers. Neuroimage. 2006;30(4):1077–87. 10.1016/j.neuroimage.2005.08.012 . 16635579

[50] Haxby JV, Gobbini MI, Furey ML, Ishai A, Schouten JL, Pietrini P. Distributed and overlapping representations of faces and objects in ventral temporal cortex. Science. 2001;293(5539):2425–30. Epub 2001/09/29. 10.1126/science.1063736 . 11577229

[51] Huth AG, Nishimoto S, Vu AT, Gallant JL. A continuous semantic space describes the representation of thousands of object and action categories across the human brain. Neuron. 2012;76(6):1210–24. 10.1016/j.neuron.2012.10.014 23259955 PMC3556488

[52] O'Toole AJ, Jiang F, Abdi H, Penard N, Dunlop JP, Parent MA. Theoretical, statistical, and practical perspectives on pattern-based classification approaches to the analysis of functional neuroimaging data. J Cogn Neurosci. 2007;19(11):1735–52. 10.1162/jocn.2007.19.11.1735 . 17958478

[53] Saxe R, Brett M, Kanwisher N. Divide and conquer: a defense of functional localizers. Neuroimage. 2006;30(4):1088–96; discussion 97–9. 10.1016/j.neuroimage.2005.12.062 . 16635578

[54] Dubois J, de Berker AO, Tsao DY. Single-unit recordings in the macaque face patch system reveal limitations of fMRI MVPA. J Neurosci. 2015;35(6):2791–802. 10.1523/JNEUROSCI.4037-14.2015 25673866 PMC4323541

[55] Haxby JV. Multivariate pattern analysis of fMRI: the early beginnings. Neuroimage. 2012;62(2):852–5. 10.1016/j.neuroimage.2012.03.016 22425670 PMC3389290

[56] Lachaux JP, Axmacher N, Mormann F, Halgren E, Crone NE. High-frequency neural activity and human cognition: past, present and possible future of intracranial EEG research. Prog Neurobiol. 2012;98(3):279–301. 10.1016/j.pneurobio.2012.06.008 22750156 PMC3980670

[57] Mukamel R, Fried I. Human intracranial recordings and cognitive neuroscience. Annu Rev Psychol. 2012;63:511–37. 10.1146/annurev-psych-120709-145401 . 21943170

[58] Tandon N. Cortical Mapping by Electrical Stimulation of Subdural Electrodes: Language areas In: Luders H, editor. Textbook of Epilepsy Surgery: Informa Healthcare; 2008 p. 1001–15.

[59] Jacques C, Witthoft N, Weiner KS, Foster BL, Rangarajan V, Hermes D, et al Corresponding ECoG and fMRI category-selective signals in human ventral temporal cortex. Neuropsychologia. 2015 10.1016/j.neuropsychologia.2015.07.024 . 26212070 PMC4724347

[60] Kadipasaoglu CM, Baboyan VG, Conner CR, Chen G, Saad ZS, Tandon N. Surface-based mixed effects multilevel analysis of grouped human electrocorticography. Neuroimage. 2014;101:215–24. 10.1016/j.neuroimage.2014.07.006 . 25019677

[61] Allison T, Puce A, Spencer DD, McCarthy G. Electrophysiological studies of human face perception. I: Potentials generated in occipitotemporal cortex by face and non-face stimuli. Cereb Cortex. 1999;9(5):415–30. Epub 1999/08/18. . 10450888 10.1093/cercor/9.5.415

[62] Davidesco I, Zion-Golumbic E, Bickel S, Harel M, Groppe DM, Keller CJ, et al Exemplar selectivity reflects perceptual similarities in the human fusiform cortex. Cereb Cortex. 2014;24(7):1879–93. 10.1093/cercor/bht038 23438448 PMC4051894

[63] Engell AD, McCarthy G. The relationship of gamma oscillations and face-specific ERPs recorded subdurally from occipitotemporal cortex. Cereb Cortex. 2011;21(5):1213–21. Epub 2010/10/22. 10.1093/cercor/bhq206 20961973 PMC3077434

[64] Engell AD, McCarthy G. Face, eye, and body selective responses in fusiform gyrus and adjacent cortex: an intracranial EEG study. Front Hum Neurosci. 2014;8:642 10.3389/fnhum.2014.00642 25191255 PMC4139958

[65] Jonas J, Descoins M, Koessler L, Colnat-Coulbois S, Sauvee M, Guye M, et al Focal electrical intracerebral stimulation of a face-sensitive area causes transient prosopagnosia. Neuroscience. 2012;222:281–8. 10.1016/j.neuroscience.2012.07.021 . 22813996

[66] Liu H, Agam Y, Madsen JR, Kreiman G. Timing, timing, timing: fast decoding of object information from intracranial field potentials in human visual cortex. Neuron. 2009;62(2):281–90. Epub 2009/05/05. 10.1016/j.neuron.2009.02.025 19409272 PMC2921507

[67] Parvizi J, Jacques C, Foster BL, Witthoft N, Rangarajan V, Weiner KS, et al Electrical stimulation of human fusiform face-selective regions distorts face perception. J Neurosci. 2012;32(43):14915–20. 10.1523/JNEUROSCI.2609-12.2012 23100414 PMC3517886

[68] Privman E, Nir Y, Kramer U, Kipervasser S, Andelman F, Neufeld MY, et al Enhanced category tuning revealed by intracranial electroencephalograms in high-order human visual areas. J Neurosci. 2007;27(23):6234–42. Epub 2007/06/08. 10.1523/JNEUROSCI.4627-06.2007 . 17553996 PMC6672161

[69] Vidal JR, Ossandon T, Jerbi K, Dalal SS, Minotti L, Ryvlin P, et al Category-Specific Visual Responses: An Intracranial Study Comparing Gamma, Beta, Alpha, and ERP Response Selectivity. Front Hum Neurosci. 2010;4:195 Epub 2011/01/27. 10.3389/fnhum.2010.00195 21267419 PMC3024557

[70] Bastin J, Vidal JR, Bouvier S, Perrone-Bertolotti M, Benis D, Kahane P, et al Temporal components in the parahippocampal place area revealed by human intracerebral recordings. J Neurosci. 2013;33(24):10123–31. 10.1523/JNEUROSCI.4646-12.2013 . 23761907 PMC6618403

[71] Argall BD, Saad ZS, Beauchamp MS. Simplified intersubject averaging on the cortical surface using SUMA. Hum Brain Mapp. 2006;27(1):14–27. Epub 2005/07/22. 10.1002/hbm.20158 . 16035046 PMC6871368

[72] Fischl B, Sereno MI, Tootell RB, Dale AM. High-resolution intersubject averaging and a coordinate system for the cortical surface. Hum Brain Mapp. 1999;8(4):272–84. Epub 2000/01/05. . 10619420 10.1002/(SICI)1097-0193(1999)8:4<272::AID-HBM10>3.0.CO;2-4 PMC6873338

[73] Saad ZS, Reynolds RC. Suma. Neuroimage. 2012;62(2):768–73. Epub 2011/09/29. 10.1016/j.neuroimage.2011.09.016 21945692 PMC3260385

[74] Damasio H, Tranel D, Grabowski T, Adolphs R, Damasio A. Neural systems behind word and concept retrieval. Cognition. 2004;92(1–2):179–229. 10.1016/j.cognition.2002.07.001 . 15037130

[75] Snodgrass JG, Vanderwart M. A standardized set of 260 pictures: norms for name agreement, image agreement, familiarity, and visual complexity. Journal of Experimental Psychology: Human Learning & Memory. 1980;6(2):174–215. 7373248 10.1037//0278-7393.6.2.174

[76] McCandliss BD, Cohen L, Dehaene S. The visual word form area: expertise for reading in the fusiform gyrus. Trends Cogn Sci. 2003;7(7):293–9. . 12860187 10.1016/s1364-6613(03)00134-7

[77] Wandell BA, Rauschecker AM, Yeatman JD. Learning to see words. Annu Rev Psychol. 2012;63:31–53. 10.1146/annurev-psych-120710-100434 21801018 PMC3228885

[78] Yeatman JD, Rauschecker AM, Wandell BA. Anatomy of the visual word form area: adjacent cortical circuits and long-range white matter connections. Brain Lang. 2013;125(2):146–55. 10.1016/j.bandl.2012.04.010 22632810 PMC3432298

[79] Conner CR, Chen G, Pieters TA, Tandon N. Category Specific Spatial Dissociations of Parallel Processes Underlying Visual Naming. Cereb Cortex. 2013 Epub 2013/05/23. 10.1093/cercor/bht130 . 23696279 PMC4153810

[80] Ellmore TM, Beauchamp MS, O'Neill TJ, Dreyer S, Tandon N. Relationships between essential cortical language sites and subcortical pathways. J Neurosurg. 2009;111(4):755–66. Epub 2009/04/21. 10.3171/2009.3.JNS081427 . 19374498

[81] Dale AM, Fischl B, Sereno MI. Cortical surface-based analysis. I. Segmentation and surface reconstruction. Neuroimage. 1999;9(2):179–94. Epub 1999/02/05. S1053-8119(98)90395-0 [pii] 10.1006/nimg.1998.0395 . 9931268

[82] Pieters TA, Conner CR, Tandon N. Recursive grid partitioning on a cortical surface model: an optimized technique for the localization of implanted subdural electrodes. J Neurosurg. 2013;118(5):1086–97. Epub 2013/03/19. 10.3171/2013.2.JNS121450 . 23495883

[83] Talairach J, Tournoux P. Co-Planar Stereotaxic Atlas of the Human Brain. New York: Theime Medical Publishers, Inc; 1988.

[84] Fischl B, Sereno M, Tootell RBH, Dale AM. High-resolution intersubject averaging and a coordinate system for the cortical surface. Hum Brain Mapp. 1999;8:272–84. 10619420 10.1002/(SICI)1097-0193(1999)8:4<272::AID-HBM10>3.0.CO;2-4 PMC6873338

[85] Holmes CJ, Hoge R, Collins L, Woods R, Toga AW, Evans AC. Enhancement of MR images using registration for signal averaging. J Comput Assist Tomogr. 1998;22(2):324–33. Epub 1998/04/08. . 9530404 10.1097/00004728-199803000-00032

[86] Anticevic A, Dierker DL, Gillespie SK, Repovs G, Csernansky JG, Van Essen DC, et al Comparing surface-based and volume-based analyses of functional neuroimaging data in patients with schizophrenia. Neuroimage. 2008;41(3):835–48. Epub 2008/04/25. 10.1016/j.neuroimage.2008.02.052 18434199 PMC2527864

[87] Dykstra AR, Chan AM, Quinn BT, Zepeda R, Keller CJ, Cormier J, et al Individualized localization and cortical surface-based registration of intracranial electrodes. Neuroimage. 2012;59(4):3563–70. Epub 2011/12/14. S1053-8119(11)01327-9 [pii] 10.1016/j.neuroimage.2011.11.046 . 22155045 PMC3288767

[88] Oosterhof NN, Wiestler T, Downing PE, Diedrichsen J. A comparison of volume-based and surface-based multi-voxel pattern analysis. Neuroimage. 2011;56(2):593–600. Epub 2010/07/14. 10.1016/j.neuroimage.2010.04.270 . 20621701

[89] Crone NE, Boatman D, Gordon B, Hao L. Induced electrocorticographic gamma activity during auditory perception. Brazier Award-winning article, 2001. Clin Neurophysiol. 2001;112(4):565–82. Epub 2001/03/29. S1388245700005459 [pii]. . 11275528 10.1016/s1388-2457(00)00545-9

[90] Fisch L, Privman E, Ramot M, Harel M, Nir Y, Kipervasser S, et al Neural "ignition": enhanced activation linked to perceptual awareness in human ventral stream visual cortex. Neuron. 2009;64(4):562–74. Epub 2009/12/01. 10.1016/j.neuron.2009.11.001 19945397 PMC2854160

[91] Rodriguez E, George N, Lachaux JP, Martinerie J, Renault B, Varela FJ. Perception's shadow: long-distance synchronization of human brain activity. Nature. 1999;397(6718):430–3. 10.1038/17120 . 9989408

[92] Crone NE, Sinai A, Korzeniewska A. High-frequency gamma oscillations and human brain mapping with electrocorticography. Prog Brain Res. 2006;159:275–95. Epub 2006/10/31. 10.1016/S0079-6123(06)59019-3 . 17071238

[93] Hermes D, Miller KJ, Wandell BA, Winawer J. Stimulus Dependence of Gamma Oscillations in Human Visual Cortex. Cereb Cortex. 2014 10.1093/cercor/bhu091 . 24855114 PMC4537439

[94] Mesgarani N, Chang EF. Selective cortical representation of attended speaker in multi-talker speech perception. Nature. 2012;485(7397):233–6. Epub 2012/04/24. 10.1038/nature11020 . 22522927 PMC3870007

[95] Miller KJ, Honey CJ, Hermes D, Rao RP, denNijs M, Ojemann JG. Broadband changes in the cortical surface potential track activation of functionally diverse neuronal populations. Neuroimage. 2014;85 Pt 2:711–20. 10.1016/j.neuroimage.2013.08.070 . 24018305 PMC4347924

[96] Manning JR, Jacobs J, Fried I, Kahana MJ. Broadband shifts in local field potential power spectra are correlated with single-neuron spiking in humans. J Neurosci. 2009;29(43):13613–20. Epub 2009/10/30. 29/43/13613 [pii] 10.1523/JNEUROSCI.2041-09.2009 19864573 PMC3001247

[97] Conner CR, Ellmore TM, Pieters TA, Disano MA, Tandon N. Variability of the Relationship between Electrophysiology and BOLD-fMRI across Cortical Regions in Humans. J Neurosci. 2011;31(36):12855–65. Epub 2011/09/09. 31/36/12855 [pii] 10.1523/JNEUROSCI.1457-11.2011 . 21900564 PMC3322193

[98] Esposito F, Singer N, Podlipsky I, Fried I, Hendler T, Goebel R. Cortex-based inter-subject analysis of iEEG and fMRI data sets: Application to sustained task-related BOLD and gamma responses. Neuroimage. 2012;66C:457–68. Epub 2012/11/10. 10.1016/j.neuroimage.2012.10.080 . 23138047

[99] He BJ, Snyder AZ, Zempel JM, Smyth MD, Raichle ME. Electrophysiological correlates of the brain's intrinsic large-scale functional architecture. Proc Natl Acad Sci U S A. 2008;105(41):16039–44. 10.1073/pnas.0807010105 18843113 PMC2564983

[100] Mukamel R, Gelbard H, Arieli A, Hasson U, Fried I, Malach R. Coupling between neuronal firing, field potentials, and FMRI in human auditory cortex. Science. 2005;309(5736):951–4. Epub 2005/08/06. 309/5736/951 [pii] 10.1126/science.1110913 . 16081741

[101] Nir Y, Fisch L, Mukamel R, Gelbard-Sagiv H, Arieli A, Fried I, et al Coupling between neuronal firing rate, gamma LFP, and BOLD fMRI is related to interneuronal correlations. Curr Biol. 2007;17(15):1275–85. Epub 2007/08/10. S0960-9822(07)01635-1 [pii] 10.1016/j.cub.2007.06.066 . 17686438

[102] Winawer J, Kay KN, Foster BL, Rauschecker AM, Parvizi J, Wandell BA. Asynchronous broadband signals are the principal source of the BOLD response in human visual cortex. Curr Biol. 2013;23(13):1145–53. 10.1016/j.cub.2013.05.001 23770184 PMC3710543

[103] Engell AD, Huettel S, McCarthy G. The fMRI BOLD signal tracks electrophysiological spectral perturbations, not event-related potentials. Neuroimage. 2012;59(3):2600–6. 10.1016/j.neuroimage.2011.08.079 21925278 PMC3277784

[104] Davidesco I, Harel M, Ramot M, Kramer U, Kipervasser S, Andelman F, et al Spatial and object-based attention modulates broadband high-frequency responses across the human visual cortical hierarchy. J Neurosci. 2013;33(3):1228–40. Epub 2013/01/18. 10.1523/JNEUROSCI.3181-12.2013 . 23325259 PMC6704891

[105] Privman E, Fisch L, Neufeld MY, Kramer U, Kipervasser S, Andelman F, et al Antagonistic relationship between gamma power and visual evoked potentials revealed in human visual cortex. Cereb Cortex. 2011;21(3):616–24. 10.1093/cercor/bhq128 . 20624838

[106] Rangarajan V, Hermes D, Foster BL, Weiner KS, Jacques C, Grill-Spector K, et al Electrical stimulation of the left and right human fusiform gyrus causes different effects in conscious face perception. J Neurosci. 2014;34(38):12828–36. 10.1523/JNEUROSCI.0527-14.2014 25232118 PMC4166163

[107] Yoshor D, Bosking WH, Ghose GM, Maunsell JH. Receptive fields in human visual cortex mapped with surface electrodes. Cereb Cortex. 2007;17(10):2293–302. 10.1093/cercor/bhl138 . 17172632

[108] Benjamini YHY. Controlling the False Discovery Rate: A Practical and Powerful Approach to Multiple Testing. Journal of the Royal Statistical Society B. 1995;57(1):289–300.

[109] Afraz SR, Kiani R, Esteky H. Microstimulation of inferotemporal cortex influences face categorization. Nature. 2006;442(7103):692–5. 10.1038/nature04982 . 16878143

[110] Ghuman AS, Brunet NM, Li Y, Konecky RO, Pyles JA, Walls SA, et al Dynamic encoding of face information in the human fusiform gyrus. Nature communications. 2014;5:5672 10.1038/ncomms6672 . 25482825 PMC4339092

[111] Green DM, Swets JA. Signal Detection Theory and Psychophysics. New York: Wiley; 1966.

[112] Matsuo T, Kawasaki K, Kawai K, Majima K, Masuda H, Murakami H, et al Alternating Zones Selective to Faces and Written Words in the Human Ventral Occipitotemporal Cortex. Cereb Cortex. 2013 10.1093/cercor/bht319 . 24285843

[113] Tang H, Buia C, Madhavan R, Crone NE, Madsen JR, Anderson WS, et al Spatiotemporal dynamics underlying object completion in human ventral visual cortex. Neuron. 2014;83(3):736–48. 10.1016/j.neuron.2014.06.017 25043420 PMC4134509

[114] Rouse AG, Williams JJ, Wheeler JJ, Moran DW. Cortical adaptation to a chronic micro-electrocorticographic brain computer interface. J Neurosci. 2013;33(4):1326–30. 10.1523/JNEUROSCI.0271-12.2013 23345208 PMC3711409

[115] Bates D. M M, B B, W S. Fitting Linear Mixed-Effects Models using lme4. Journal of Statistical Software. 2015.

[116] Bates DM, M.; Bolker, B.; Walker, S. lme4: Linear mixed-effects models using Eigen and S4. R package version 11–8. 2015.

[117] Kuznetsova AB, B.; Christensen, H.B;. lmerTest: Tests in Linear Mixed Effects Modles. R package version 20–29. 2015.

[118] Baayen RH, D DJ, M B, D.. Mixed-effects modeling with crossed random effects for subjects and items. Journal of memory and language. 2008:390–412. 10.1016/j.jml.2007.12.005

[119] Falk EB, O'Donnell MB, Cascio CN, Tinney F, Kang Y, Lieberman MD, et al Self-affirmation alters the brain's response to health messages and subsequent behavior change. Proc Natl Acad Sci U S A. 2015;112(7):1977–82. 10.1073/pnas.1500247112 25646442 PMC4343089

[120] R Core Team. R: A Language and Environment for Statistical Computing. R Foundation for Statistical Computing; 2012.

[121] Wickham H. ggplot2: elegant graphics for data analysis: Springer New York; 2009.

[122] Kadipasaoglu CM, Forseth K, Whaley M, Conner CR, Rollo MJ, Baboyan VG, et al Development of grouped icEEG for the study of cognitive processing. Frontiers in psychology. 2015;6:1008 10.3389/fpsyg.2015.01008 26257673 PMC4508923

[123] Pourtois G, Peelen MV, Spinelli L, Seeck M, Vuilleumier P. Direct intracranial recording of body-selective responses in human extrastriate visual cortex. Neuropsychologia. 2007;45(11):2621–5. 10.1016/j.neuropsychologia.2007.04.005 . 17499819

[124] Weiner KS, Grill-Spector K. Not one extrastriate body area: using anatomical landmarks, hMT+, and visual field maps to parcellate limb-selective activations in human lateral occipitotemporal cortex. Neuroimage. 2011;56(4):2183–99. 10.1016/j.neuroimage.2011.03.041 21439386 PMC3138128

[125] Grill-Spector K, Kourtzi Z, Kanwisher N. The lateral occipital complex and its role in object recognition. Vision Res. 2001;41(10–11):1409–22. . 11322983 10.1016/s0042-6989(01)00073-6

[126] Lingnau A, Downing PE. The lateral occipitotemporal cortex in action. Trends Cogn Sci. 2015;19(5):268–77. 10.1016/j.tics.2015.03.006 . 25843544

[127] Price CJ, Devlin JT. The interactive account of ventral occipitotemporal contributions to reading. Trends Cogn Sci. 2011;15(6):246–53. 10.1016/j.tics.2011.04.001 21549634 PMC3223525

[128] Bentin S, Allison T, Puce A, Perez E, McCarthy G. Electrophysiological Studies of Face Perception in Humans. J Cogn Neurosci. 1996;8(6):551–65. 10.1162/jocn.1996.8.6.551 20740065 PMC2927138

[129] Caramazza A, Mahon BZ. The organization of conceptual knowledge: the evidence from category-specific semantic deficits. Trends Cogn Sci. 2003;7(8):354–61. Epub 2003/08/09. . 12907231 10.1016/s1364-6613(03)00159-1

[130] Chan AM, Baker JM, Eskandar E, Schomer D, Ulbert I, Marinkovic K, et al First-pass selectivity for semantic categories in human anteroventral temporal lobe. J Neurosci. 2011;31(49):18119–29. 10.1523/JNEUROSCI.3122-11.2011 22159123 PMC3286838

[131] Drane DL, Ojemann GA, Aylward E, Ojemann JG, Johnson LC, Silbergeld DL, et al Category-specific naming and recognition deficits in temporal lobe epilepsy surgical patients. Neuropsychologia. 2008;46(5):1242–55. 10.1016/j.neuropsychologia.2007.11.034 18206185 PMC2474808

[132] Gaillard R, Naccache L, Pinel P, Clemenceau S, Volle E, Hasboun D, et al Direct intracranial, FMRI, and lesion evidence for the causal role of left inferotemporal cortex in reading. Neuron. 2006;50(2):191–204. Epub 2006/04/25. 10.1016/j.neuron.2006.03.031 . 16630832

[133] Gauthier I, Skudlarski P, Gore JC, Anderson AW. Expertise for cars and birds recruits brain areas involved in face recognition. Nat Neurosci. 2000;3(2):191–7. . 10649576 10.1038/72140

[134] Kanwisher N. Functional specificity in the human brain: a window into the functional architecture of the mind. Proc Natl Acad Sci U S A. 2010;107(25):11163–70. Epub 2010/05/21. 10.1073/pnas.1005062107 20484679 PMC2895137

[135] Kojima K, Brown EC, Matsuzaki N, Asano E. Animal category-preferential gamma-band responses in the lower- and higher-order visual areas: intracranial recording in children. Clin Neurophysiol. 2013;124(12):2368–77. 10.1016/j.clinph.2013.05.030 23910987 PMC3834016

[136] Martin A. The representation of object concepts in the brain. Annu Rev Psychol. 2007;58:25–45. Epub 2006/09/14. 10.1146/annurev.psych.57.102904.190143 . 16968210

[137] Puce A, Allison T, McCarthy G. Electrophysiological studies of human face perception. III: Effects of top-down processing on face-specific potentials. Cereb Cortex. 1999;9(5):445–58. Epub 1999/08/18. . 10450890 10.1093/cercor/9.5.445

[138] Capitani E, Laiacona M, Mahon B, Caramazza A. What are the facts of semantic category-specific deficits? A critical review of the clinical evidence. Cognitive neuropsychology. 2003;20(3):213–61. 10.1080/02643290244000266 . 20957571

[139] Marr D. Vision: The MIT Press; 1982.

[140] Kravitz DJ, Saleem KS, Baker CI, Ungerleider LG, Mishkin M. The ventral visual pathway: an expanded neural framework for the processing of object quality. Trends Cogn Sci. 2013;17(1):26–49. 10.1016/j.tics.2012.10.011 23265839 PMC3532569

[141] Rossion B. Understanding face perception by means of prosopagnosia and neuroimaging. Frontiers in bioscience. 2014;6:258–307. . 24896206 10.2741/E706

[142] McCarthy G, Puce A, Belger A, Allison T. Electrophysiological studies of human face perception. II: Response properties of face-specific potentials generated in occipitotemporal cortex. Cereb Cortex. 1999;9(5):431–44. Epub 1999/08/18. . 10450889 10.1093/cercor/9.5.431

[143] Rossion B. Understanding face perception by means of human electrophysiology. Trends Cogn Sci. 2014;18(6):310–8. 10.1016/j.tics.2014.02.013 . 24703600

[144] Weiner KS, Grill-Spector K. Sparsely-distributed organization of face and limb activations in human ventral temporal cortex. Neuroimage. 2010;52(4):1559–73. 10.1016/j.neuroimage.2010.04.262 20457261 PMC3122128

[145] Peelen MV, Downing PE. The neural basis of visual body perception. Nat Rev Neurosci. 2007;8(8):636–48. 10.1038/nrn2195 . 17643089

[146] Cohen L, Dehaene S. Specialization within the ventral stream: the case for the visual word form area. Neuroimage. 2004;22(1):466–76. . 15110040 10.1016/j.neuroimage.2003.12.049

[147] Avidan G, Harel M, Hendler T, Ben-Bashat D, Zohary E, Malach R. Contrast sensitivity in human visual areas and its relationship to object recognition. J Neurophysiol. 2002;87(6):3102–16. . 12037211 10.1152/jn.2002.87.6.3102

[148] Davidenko N, Remus DA, Grill-Spector K. Face-likeness and image variability drive responses in human face-selective ventral regions. Hum Brain Mapp. 2012;33(10):2334–49. 10.1002/hbm.21367 21823208 PMC3404198

[149] Grill-Spector K, Kushnir T, Edelman S, Itzchak Y, Malach R. Cue-invariant activation in object-related areas of the human occipital lobe. Neuron. 1998;21(1):191–202. . 9697863 10.1016/s0896-6273(00)80526-7

[150] Kourtzi Z, Kanwisher N. Representation of perceived object shape by the human lateral occipital complex. Science. 2001;293(5534):1506–9. 10.1126/science.1061133 . 11520991

[151] Mendola JD, Dale AM, Fischl B, Liu AK, Tootell RB. The representation of illusory and real contours in human cortical visual areas revealed by functional magnetic resonance imaging. J Neurosci. 1999;19(19):8560–72. . 10493756 10.1523/JNEUROSCI.19-19-08560.1999 PMC6783043

[152] Moutoussis K, Zeki S. The relationship between cortical activation and perception investigated with invisible stimuli. Proc Natl Acad Sci U S A. 2002;99(14):9527–32. 10.1073/pnas.142305699 12089336 PMC123174

[153] Vinberg J, Grill-Spector K. Representation of shapes, edges, and surfaces across multiple cues in the human visual cortex. J Neurophysiol. 2008;99(3):1380–93. 10.1152/jn.01223.2007 . 18171705

[154] Walther DB, Chai B, Caddigan E, Beck DM, Fei-Fei L. Simple line drawings suffice for functional MRI decoding of natural scene categories. Proc Natl Acad Sci U S A. 2011;108(23):9661–6. 10.1073/pnas.1015666108 21593417 PMC3111263

[155] Yamins DL, DiCarlo JJ. Eight open questions in the computational modeling of higher sensory cortex. Curr Opin Neurobiol. 2016;37:114–20. 10.1016/j.conb.2016.02.001 . 26921828

[156] Andrews TJ, Clarke A, Pell P, Hartley T. Selectivity for low-level features of objects in the human ventral stream. Neuroimage. 2010;49(1):703–11. 10.1016/j.neuroimage.2009.08.046 . 19716424

[157] Baldassi C, Alemi-Neissi A, Pagan M, Dicarlo JJ, Zecchina R, Zoccolan D. Shape similarity, better than semantic membership, accounts for the structure of visual object representations in a population of monkey inferotemporal neurons. PLoS Comput Biol. 2013;9(8):e1003167 10.1371/journal.pcbi.1003167 23950700 PMC3738466

[158] Rice GE, Watson DM, Hartley T, Andrews TJ. Low-level image properties of visual objects predict patterns of neural response across category-selective regions of the ventral visual pathway. J Neurosci. 2014;34(26):8837–44. 10.1523/JNEUROSCI.5265-13.2014 24966383 PMC4069357

[159] Majaj NJ, Hong H, Solomon EA, DiCarlo JJ. Simple Learned Weighted Sums of Inferior Temporal Neuronal Firing Rates Accurately Predict Human Core Object Recognition Performance. J Neurosci. 2015;35(39):13402–18. 10.1523/JNEUROSCI.5181-14.2015 26424887 PMC4588611

[160] Yamins DL, Hong H, Cadieu CF, Solomon EA, Seibert D, DiCarlo JJ. Performance-optimized hierarchical models predict neural responses in higher visual cortex. Proc Natl Acad Sci U S A. 2014;111(23):8619–24. 10.1073/pnas.1403112111 24812127 PMC4060707

[161] Lehky SR, Tanaka K. Neural representation for object recognition in inferotemporal cortex. Curr Opin Neurobiol. 2016;37:23–35. 10.1016/j.conb.2015.12.001 . 26771242

[162] Mahon BZ, Anzellotti S, Schwarzbach J, Zampini M, Caramazza A. Category-specific organization in the human brain does not require visual experience. Neuron. 2009;63(3):397–405. Epub 2009/08/15. 10.1016/j.neuron.2009.07.012 19679078 PMC2743253

[163] Khaligh-Razavi SM, Kriegeskorte N. Deep supervised, but not unsupervised, models may explain IT cortical representation. PLoS Comput Biol. 2014;10(11):e1003915 10.1371/journal.pcbi.1003915 25375136 PMC4222664

[164] Connolly AC, Guntupalli JS, Gors J, Hanke M, Halchenko YO, Wu YC, et al The representation of biological classes in the human brain. J Neurosci. 2012;32(8):2608–18. 10.1523/JNEUROSCI.5547-11.2012 22357845 PMC3532035

[165] Kaiser D, Azzalini DC, Peelen MV. Shape-independent object category responses revealed by MEG and fMRI decoding. J Neurophysiol. 2016:jn 01074 2015. 10.1152/jn.01074.2015 . 26740535 PMC4869498

