# Supplementary figures and images for: Category-Selectivity in Human Visual Cortex Follows Cortical Topology: A Grouped icEEG Study
Source: PLoS One. 2016 Jun 7;11(6):e0157109. doi: 10.1371/journal.pone.0157109 (PMC4896492; doi:10.1371/journal.pone.0157109)

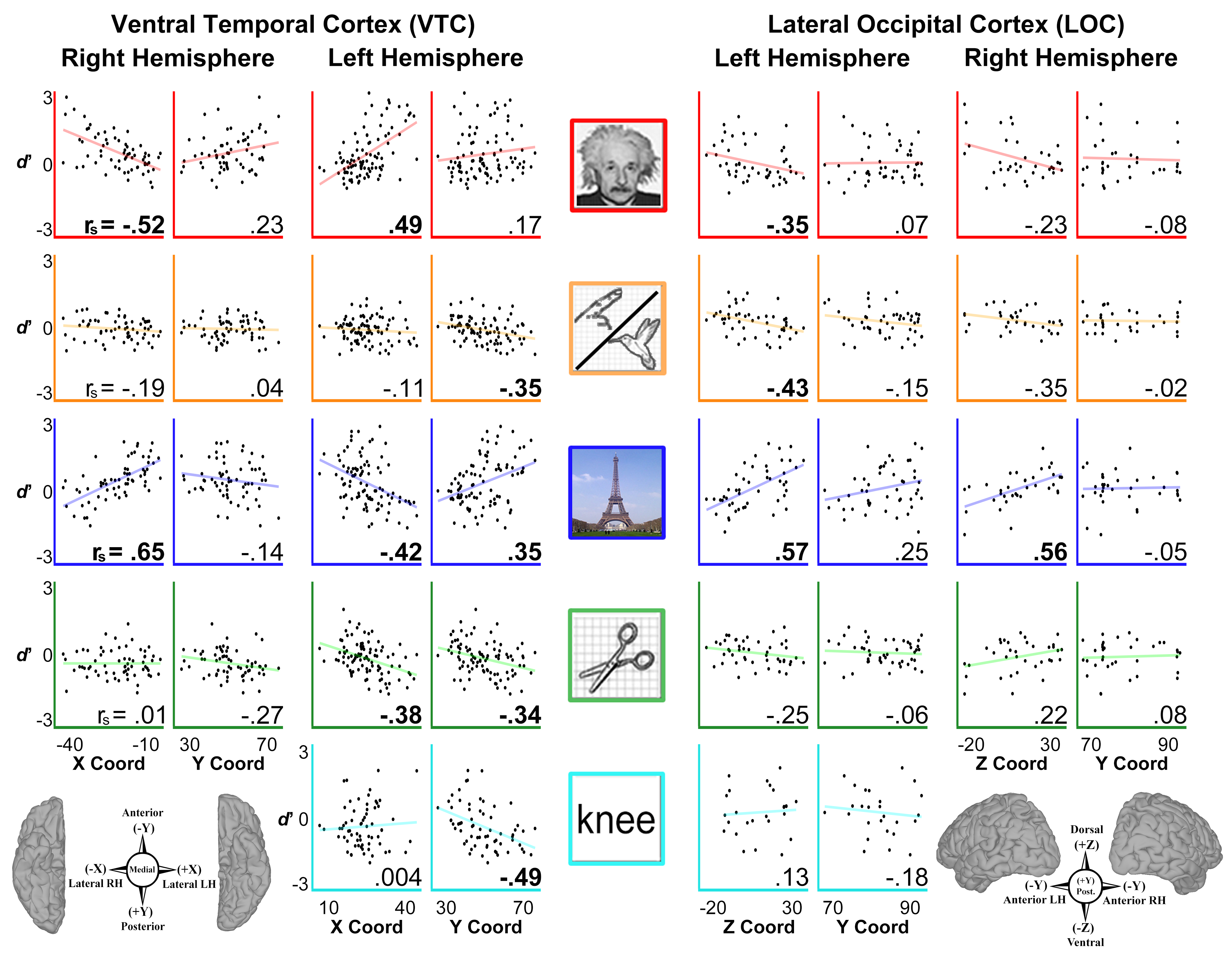

Supplement: S1 Fig — Scatterplots depict grouped d’ indices for each category plotted vs. subdural electrode (SDE) coordinates (in Talairach space) per hemisphere in each region. In the ventral temporal cortex (VTC; RH n = 64, LH n = 94), comparisons were made against the x and y coordinates. In the lateral occipital cortex (LOC; LH n = 48, RH n = 35), comparisons were made with the z and y coordinates. For each plot, regression lines were fitted (color-coded by category), and the strengths of association were estimated using Spearman correlations (bottom right, bold text denotes FDR corrected q ≤ 0.05, for multiple comparison across categories and SDEs per region and hemisphere). Spearman correlations were selected (over Pearson’s) for their robustness to outlier influence and smaller sample sizes. Furthermore, Spearman’s correlations test for monotonic relationships, and the relationships between d’ indices and SDE coordinates are not known a priori to be linear. (TIF) [file pone.0157109.s004.tif]

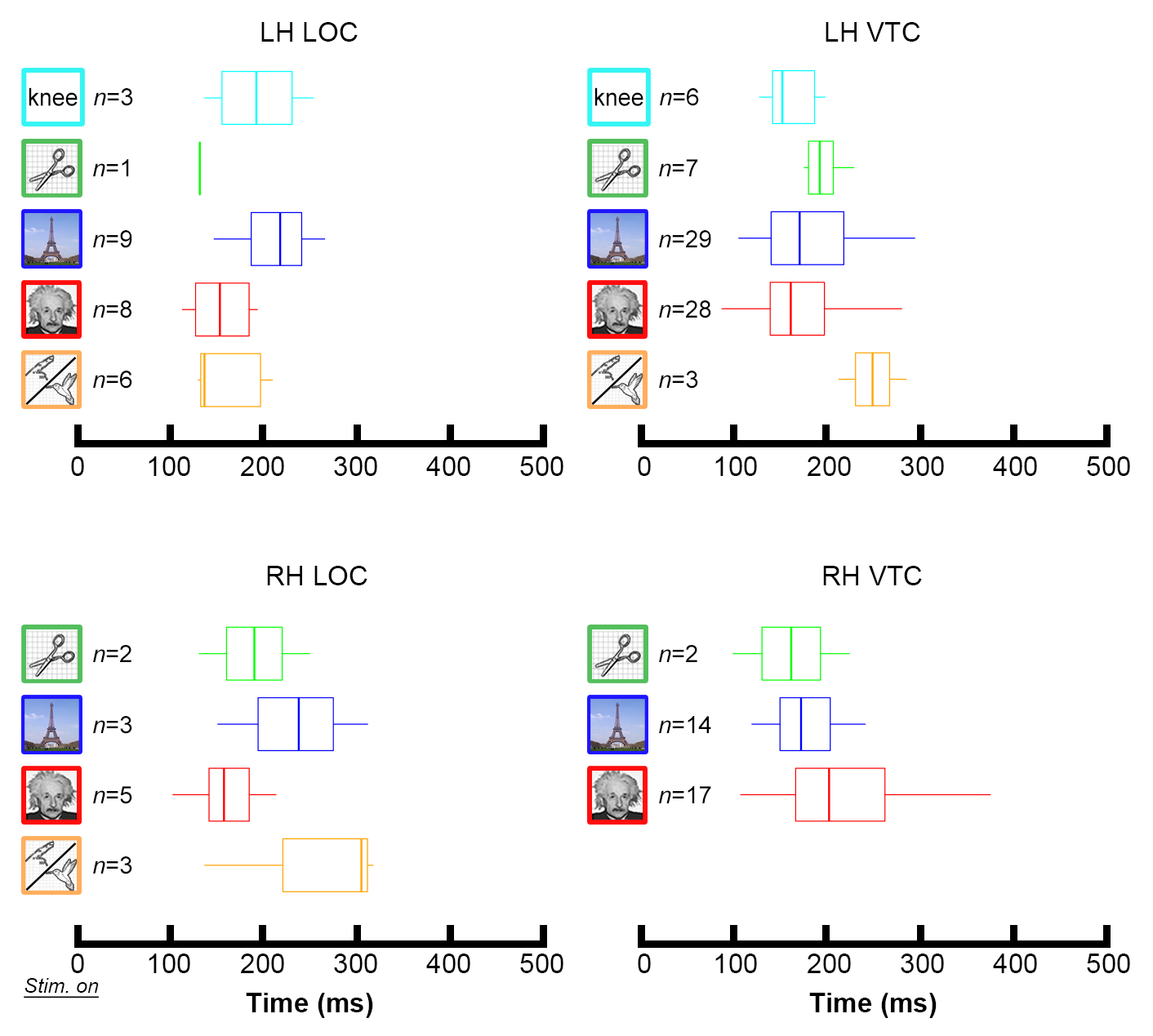

Supplement: S2 Fig — Box plots depict median onset latency of category-selectivity in the left (LH) and right (RH) hemisphere lateral occipital cortex (LOC) and ventral temporal cortex (VTC), for the five categories of interest: words (cyan), tools (green), places (blue), faces (red) and non-face animate (body-parts and animals) stimuli. Timing of the onset of selectivity was evaluated for each category-selectivity SDE per subject, and determined using pairwise comparisons in broadband gamma activity time-series, for each category against all others. No significant differences between categories (following corrections for multiple comparisons) were noted, although low sample sizes likely underpowered these contrasts. Word stimuli were not tested in the right hemisphere, and in the RH VTC, no significant animate SDEs were observed. (TIF) [file pone.0157109.s005.tif]

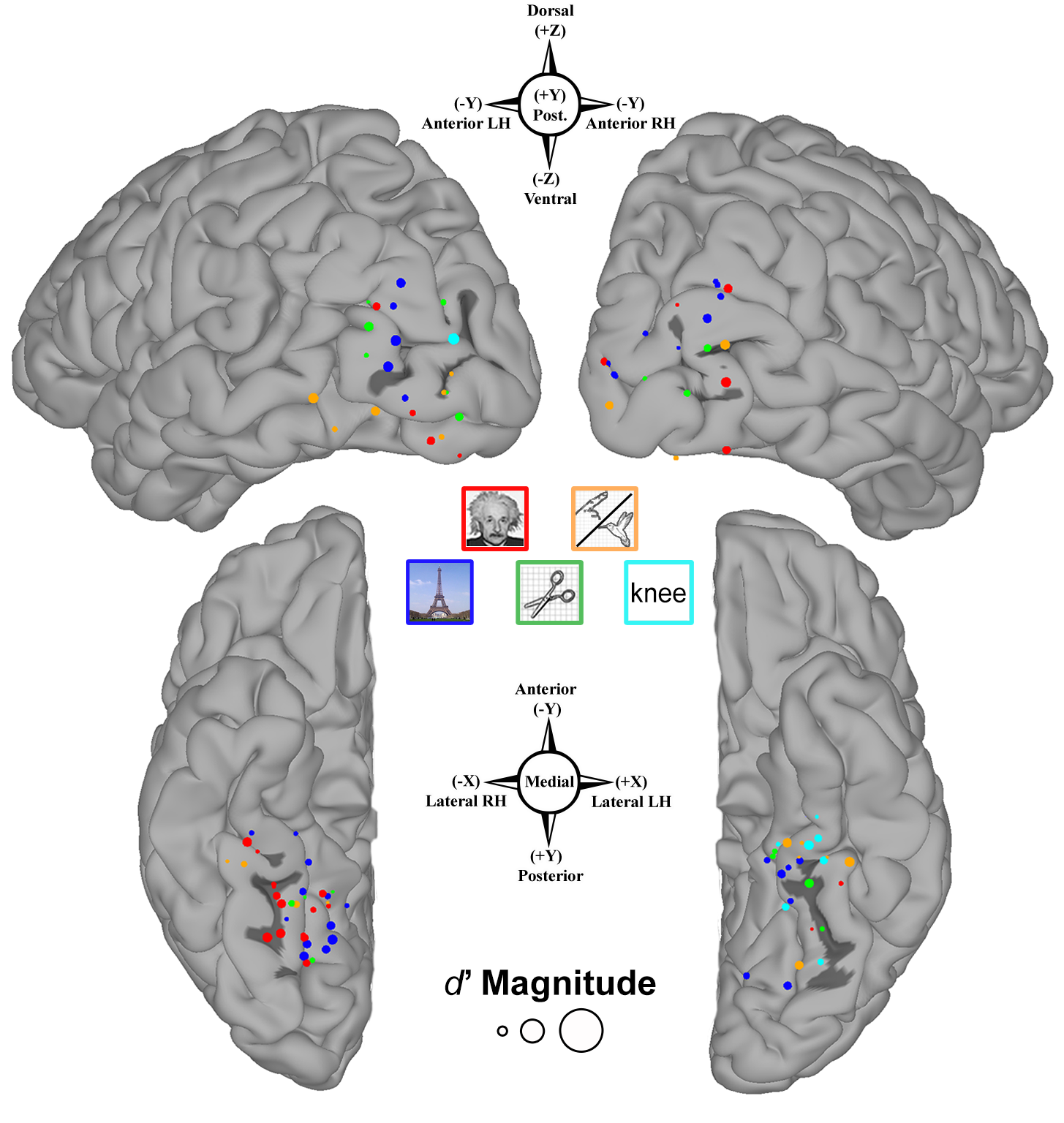

Supplement: S3 Fig — Non category-selective subdural electrodes (SDEs) are visualized on the MNI N27 template brain (aligned to Talairach coordinate space) after surface based normalization. SDEs are color-coded by the category with the largest d’ index for that electrode (matched to image legends). SDE diameter reflects the magnitude of the d’ value for that category, scaled by the largest d’ value across categories per region (regions per hemisphere are scaled differently). Compass points denote SDE coordinates (Talairach space) and direction. Notably, in the postero-lateral aspects of bilateral ventral temporal cortex, no non-significant SDEs are observed. (TIF) [file pone.0157109.s006.tif]
